# Supplementary material for: Synthesis, Cytotoxicity and Molecular Docking of New Hybrid Compounds by Combination of Curcumin with Oleanolic Acid
Source: Biomedicines. 2023 May 23;11(6):1506. doi: 10.3390/biomedicines11061506 (PMC10295546; doi:10.3390/biomedicines11061506)
Supplement: Supplementary file 1 [file biomedicines-11-01506-s001.zip › biomedicines-2350219-supplementary.pdf]

Supplementary Material

Synthesis, Cytotoxicity and Molecular Docking of New Hybrid Compounds by Combination of Curcumin with Oleanolic Acid

Katarzyna Sowa-Kasprzak[1*], Ewa Totoń[2], Jacek Kujawski[1], Dorota Olender[1], Natalia Lisiak[2], Lucjusz Zaprutko[1] Błażej Rubiś[2], Mariusz Kaczmarek[3], Anna Pawełczyk[1]

[1] Chair and Department of Organic Chemistry, Poznan University of Medical Sciences, Grunwaldzka 6 Str., 60-780 Poznań, Poland
[2] Department of Clinical Chemistry and Molecular Diagnostics, Poznan University of Medical Sciences, Rokietnicka 3 Str., 60-806 Poznań, Poland; etoton@ump.edu.pl (E.T.)
[3] Department of Cancer Diagnostics and Immunology, Gene Therapy Unit, Greater Poland Cancer Centre, Garbary 15 Str., 61-866, Poznań, Poland
* Correspondence: kasik@ump.edu.pl (K.S.K.)

Table of Contents

Contents **Page No.**

1. The first poses of the docked ligands to compounds **KS1–KS8**
- 4kik.pdb protein (Figure S1-S9) S2
- 2w96.pdb protein (Figure S10-S18) S11
- 3dt3.pdb protein (Figure S19-S26) S20
- 1l2j.pdb protein (Figure S27-S35) S29

2. ^{1}H-NMR and ^{13}C-NMR of the synthesized compounds S38

KS1

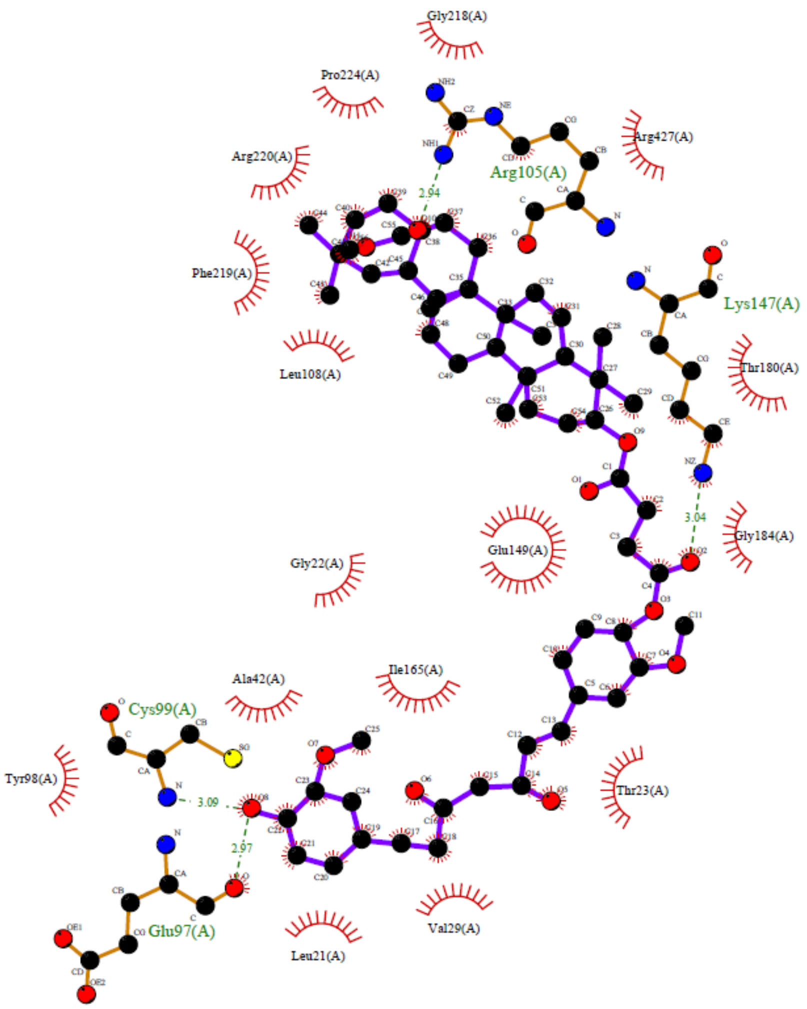

Figure S1 The first poses of the docked ligands to compound **KS1**; hydrogen atoms are omitted; 4kik.pdb protein (LigPlot+ v.2.2 software).

KS2_keto

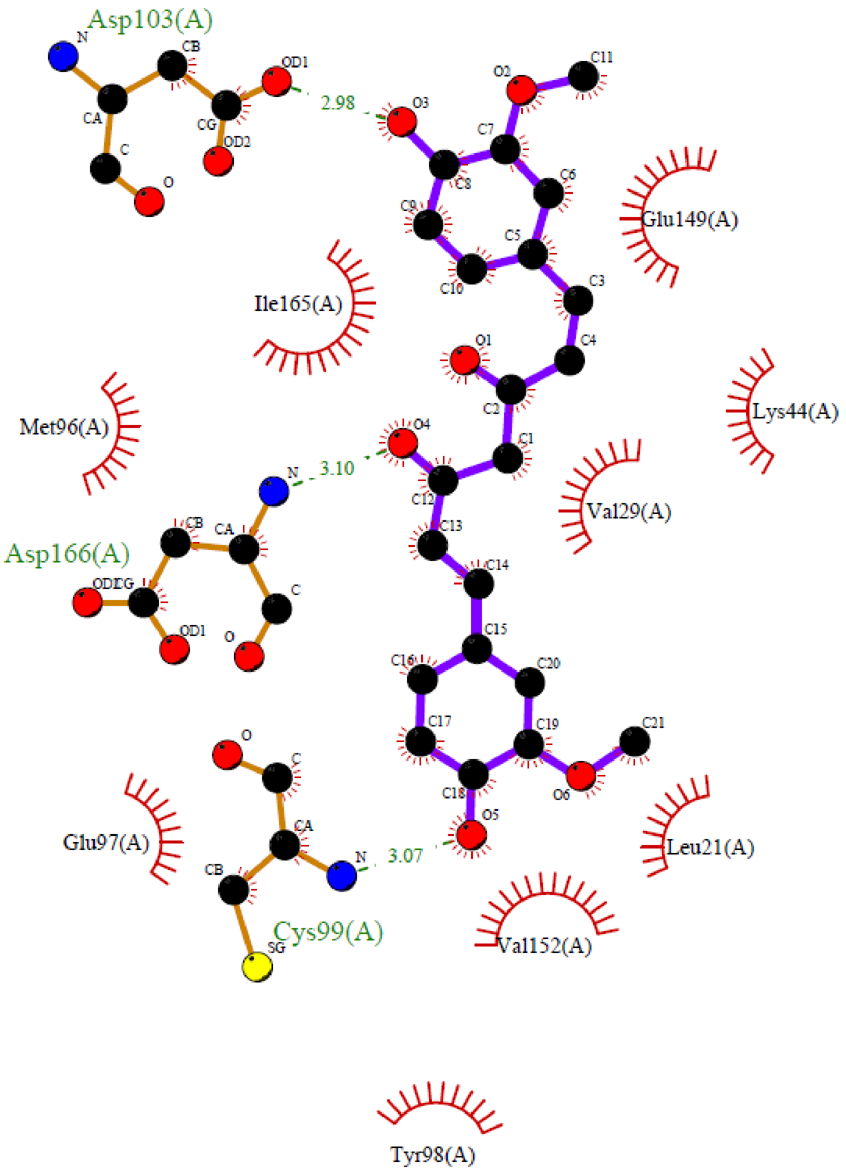

Figure S2 The first poses of the docked ligands to compound **KS2_keto**; hydrogen atoms are omitted; 4kik.pdb protein (LigPlot+ v.2.2 software).

KS2_enol

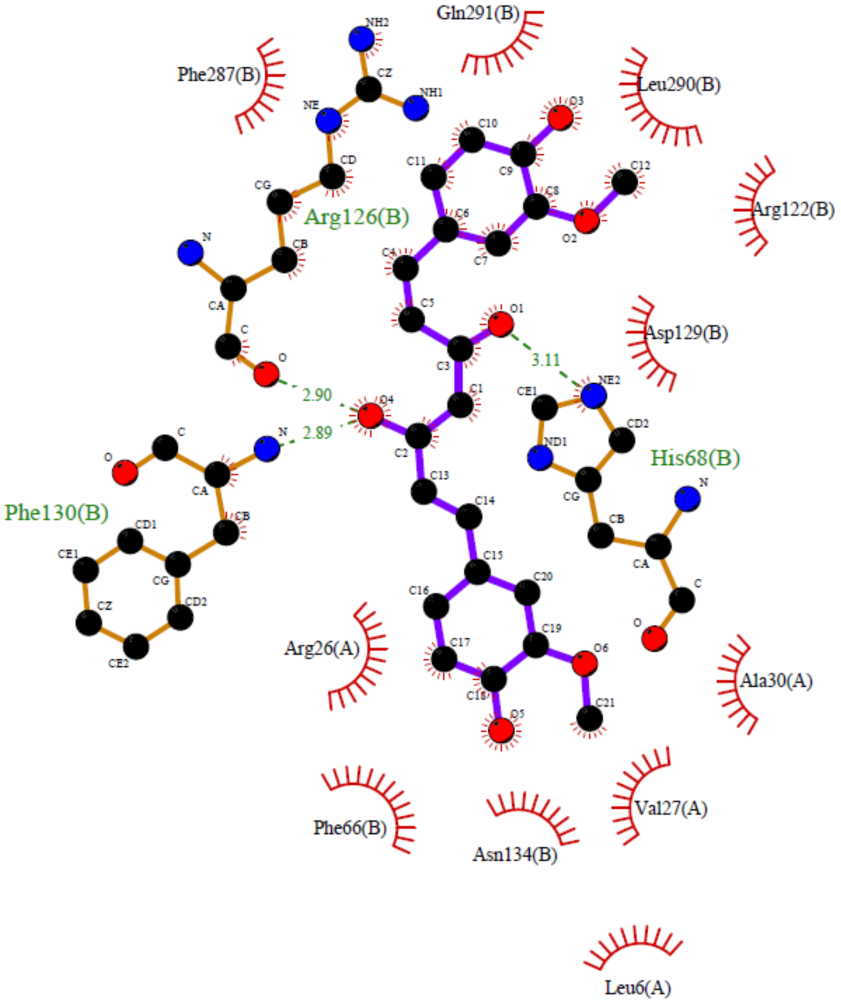

Figure S3 The first poses of the docked ligands to compound **KS2_enol**; hydrogen atoms are omitted; 4kik.pdb protein (LigPlot+ v.2.2 software).

KS3

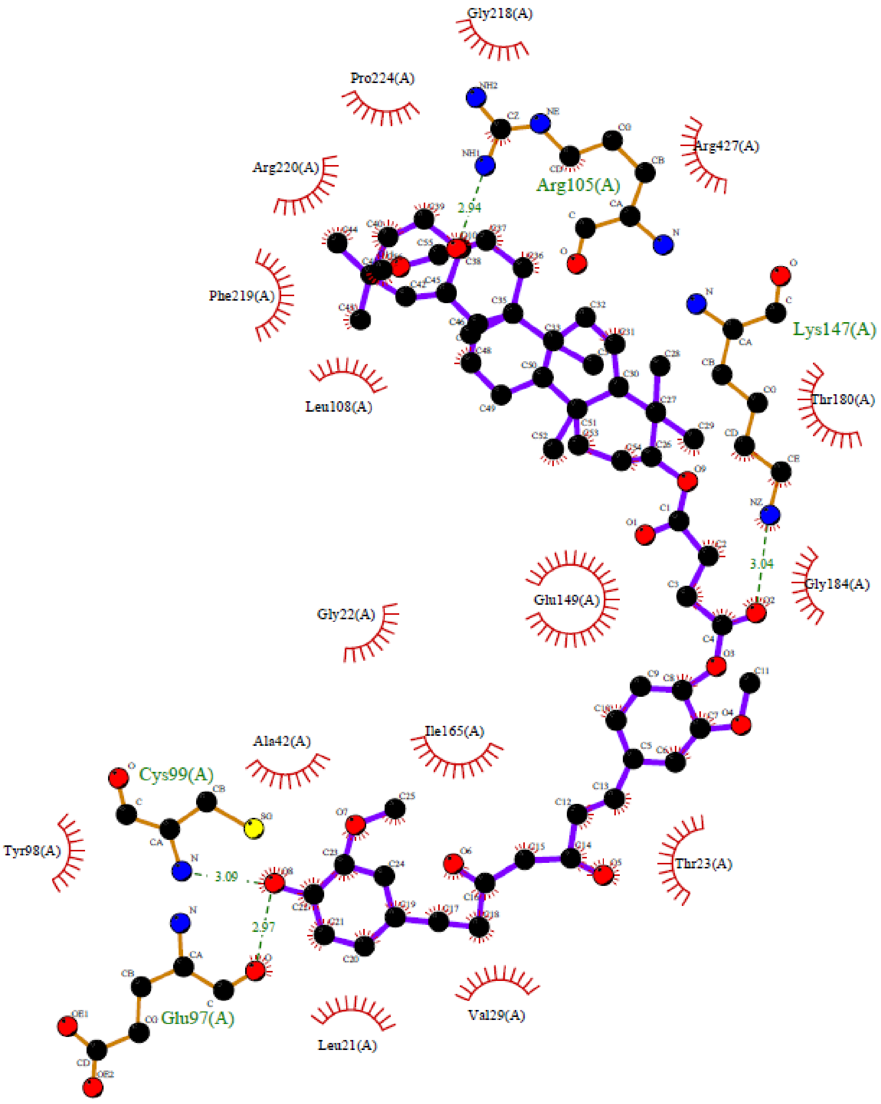

Figure S4 The first poses of the docked ligands to compound **KS3**; hydrogen atoms are omitted; 4kik.pdb protein (LigPlot+ v.2.2 software).

KS4

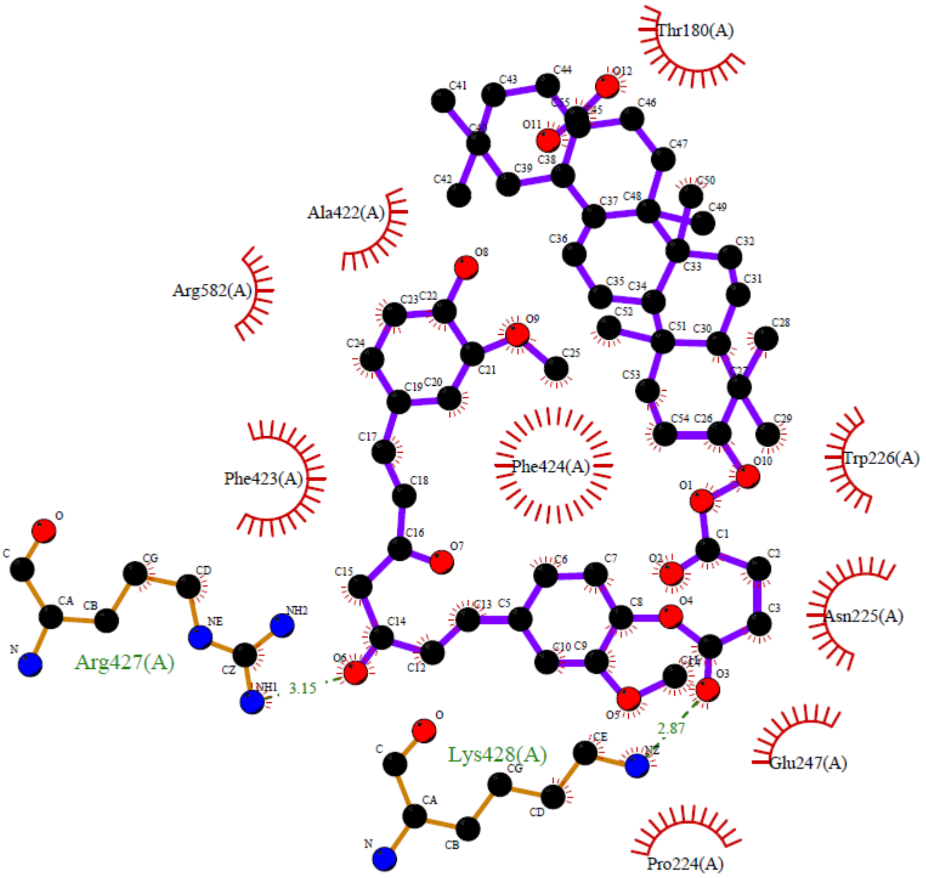

Figure S5 The first poses of the docked ligands to compound **KS4**; hydrogen atoms are omitted; 4kik.pdb protein (LigPlot+ v.2.2 software).

KS5

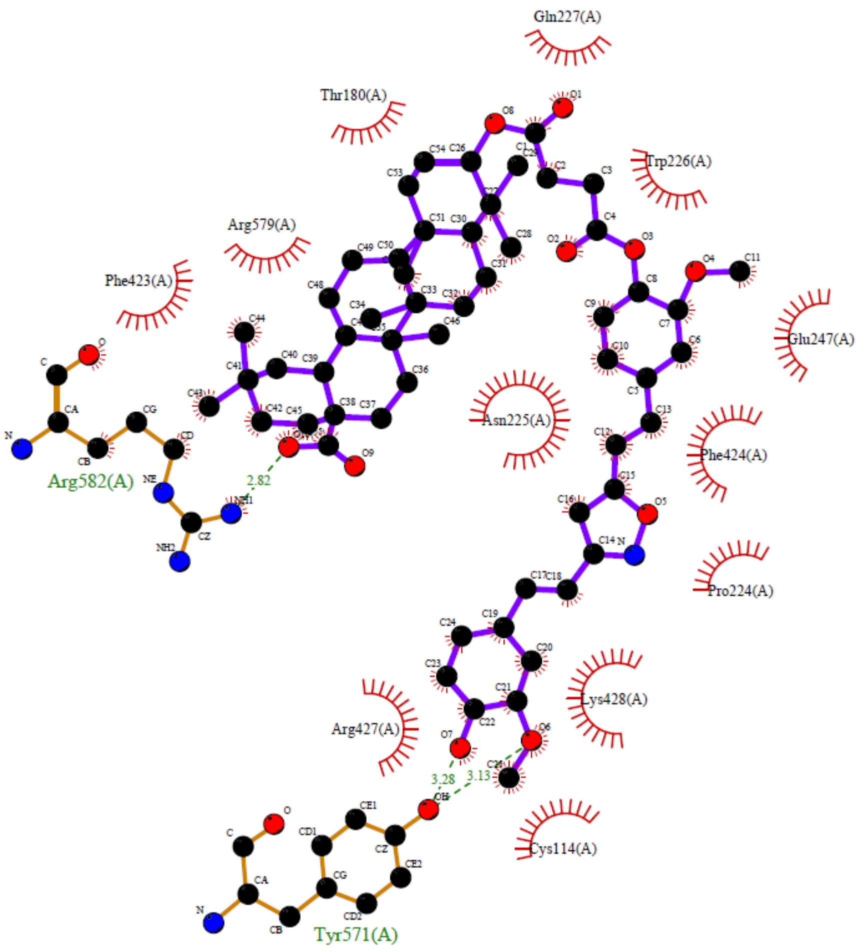

Figure S6 The first poses of the docked ligands to compound **KS5**; hydrogen atoms are omitted; 4kik.pdb protein (LigPlot+ v.2.2 software).

KS6

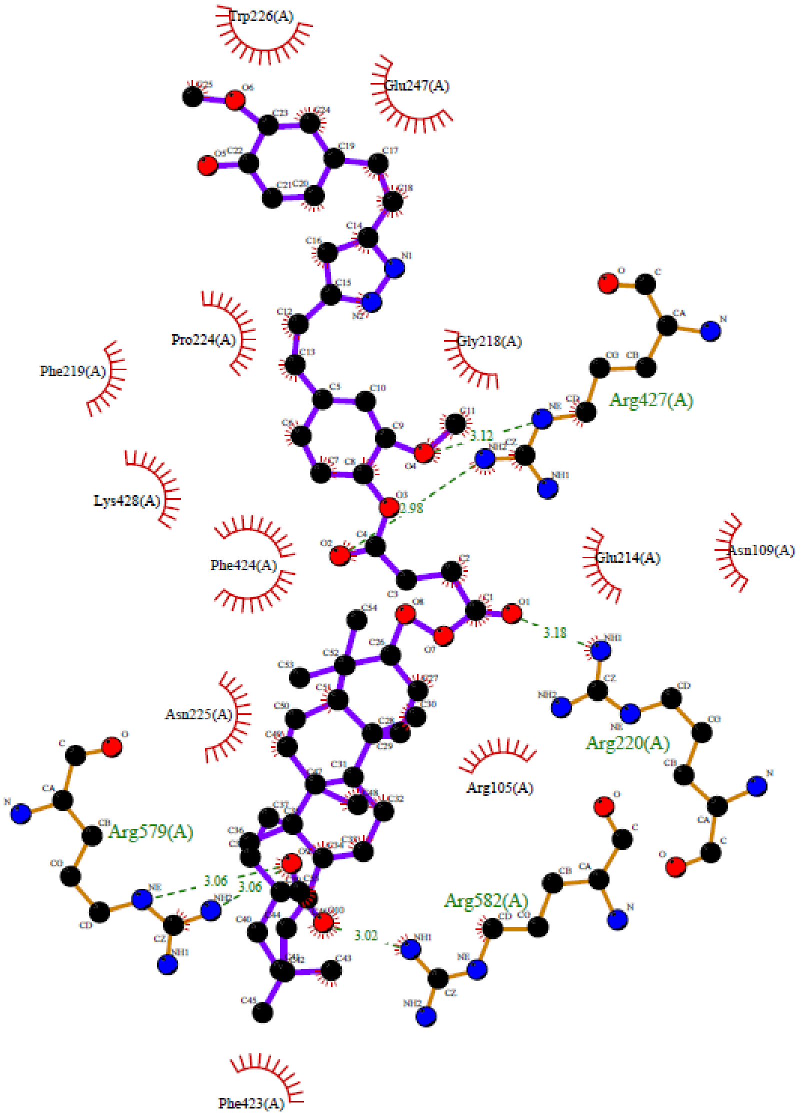

Figure S7 The first poses of the docked ligands to compound **KS6**; hydrogen atoms are omitted; 4kik.pdb protein (LigPlot+ v.2.2 software).

KS7

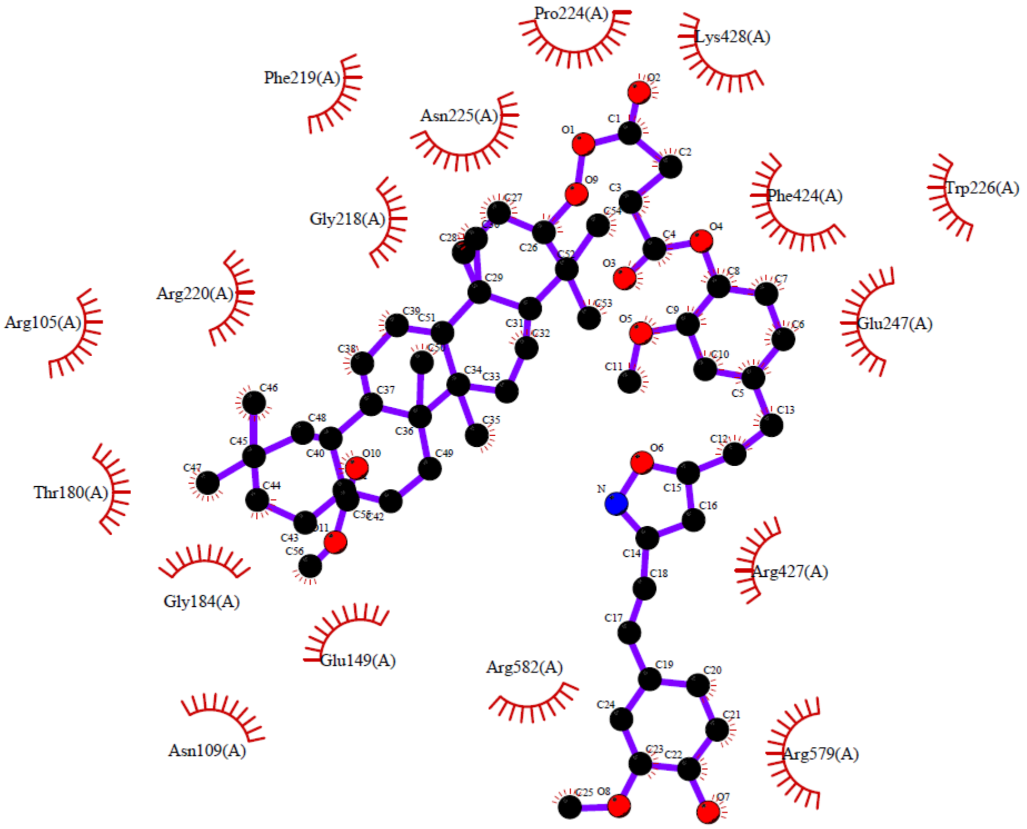

Figure S8 The first poses of the docked ligands to compound **KS7**; hydrogen atoms are omitted; 4kik.pdb protein (LigPlot+ v.2.2 software).

KS8

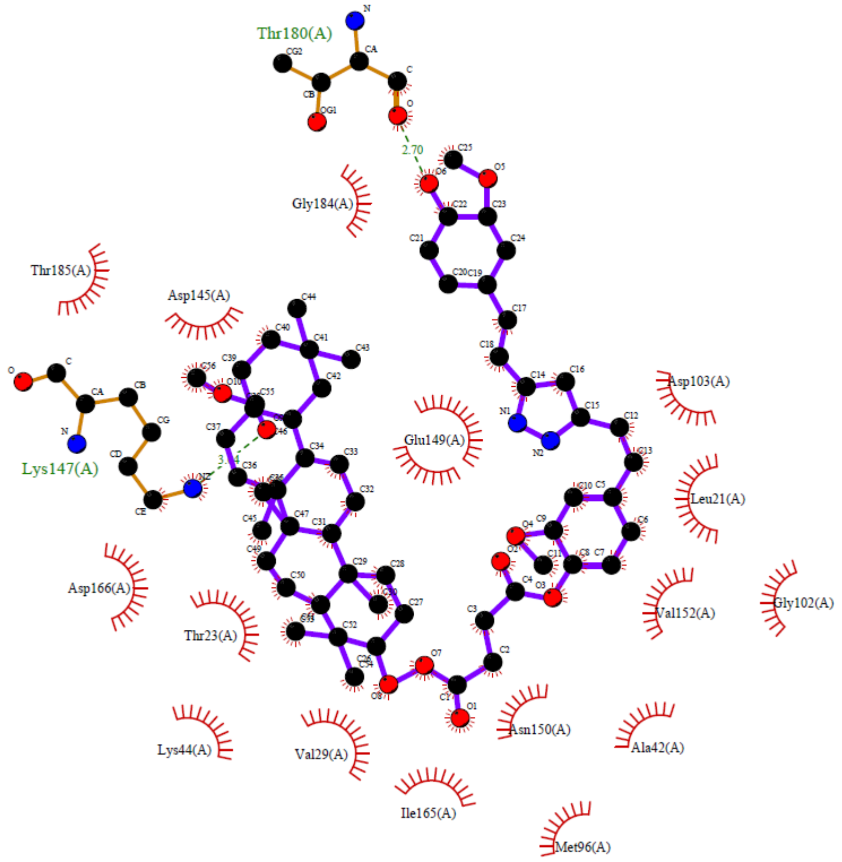

Figure S9 The first poses of the docked ligands to compound **KS8**; hydrogen atoms are omitted; 4kik.pdb protein (LigPlot+ v.2.2 software).

KS1

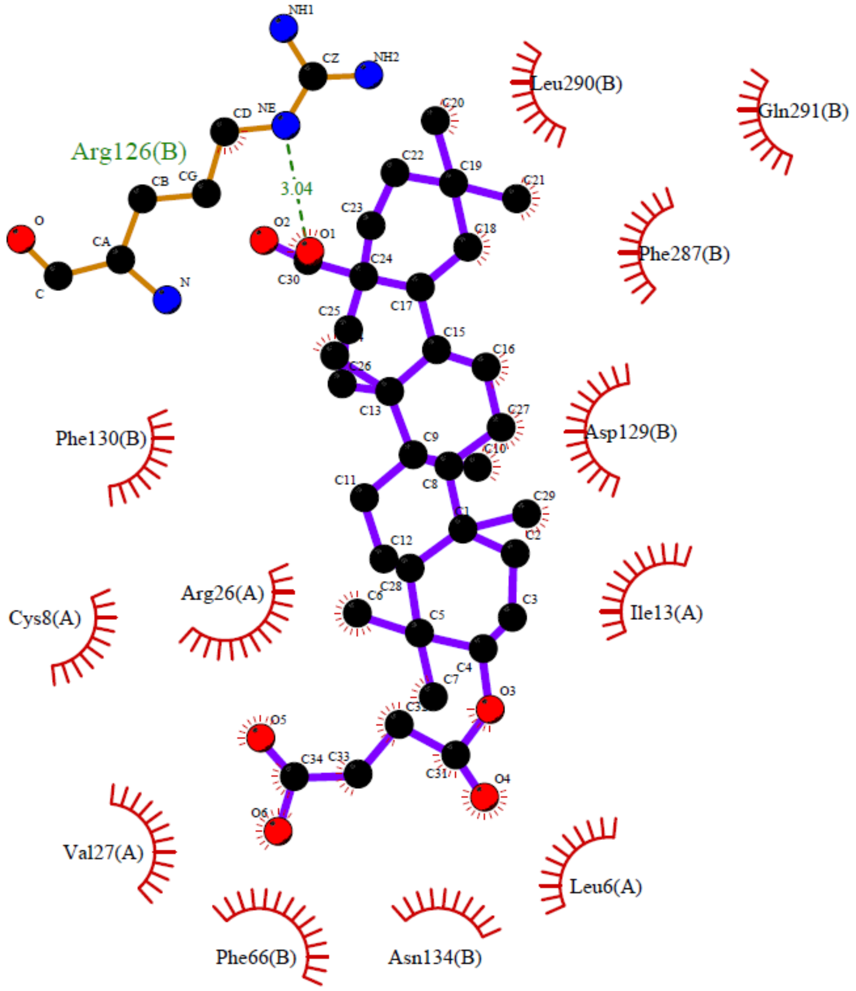

Figure S10 The first poses of the docked ligands to compound **KS1**; hydrogen atoms are omitted; 2w96.pdb protein (LigPlot+ v.2.2 software).

KS2_keto

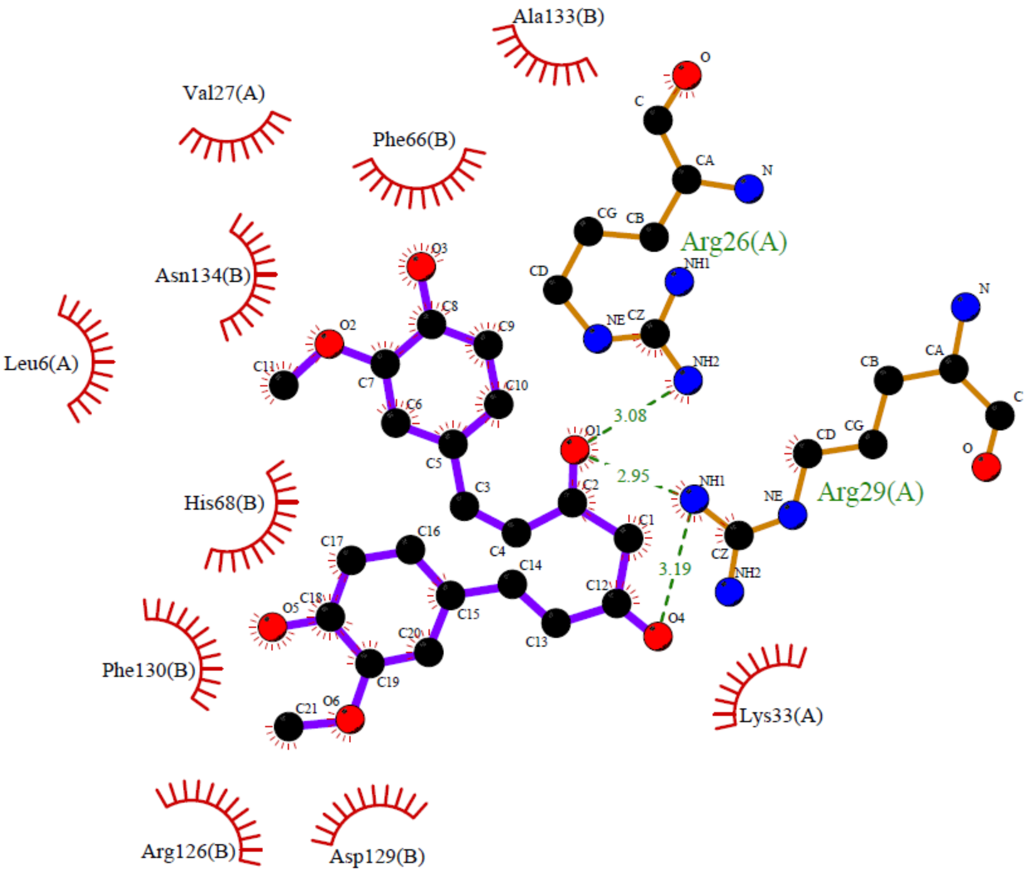

Figure S11 The first poses of the docked ligands to compound **KS2_keto**; hydrogen atoms are omitted; 2w96.pdb protein (LigPlot+ v.2.2 software).

KS2_enol

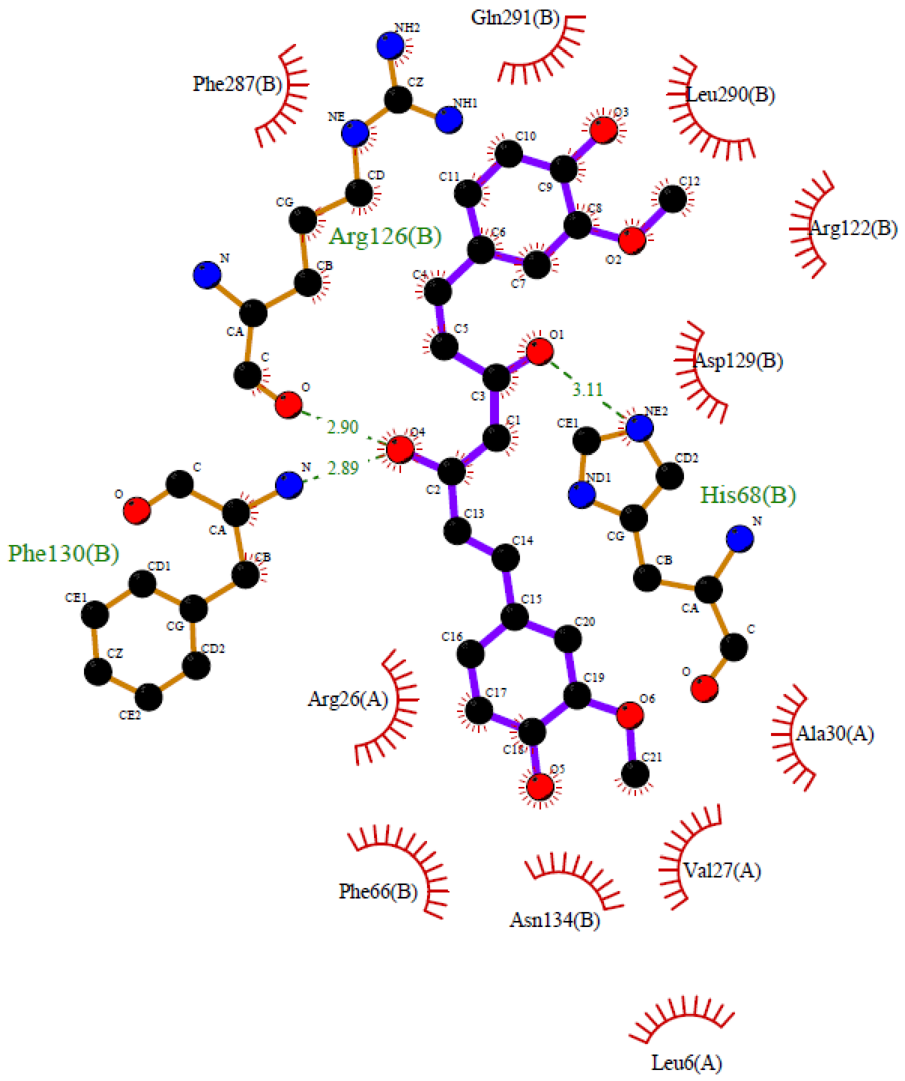

Figure S12 The first poses of the docked ligands to compound **KS2_enol**; hydrogen atoms are omitted; 2w96.pdb protein (LigPlot+ v.2.2 software).

KS3

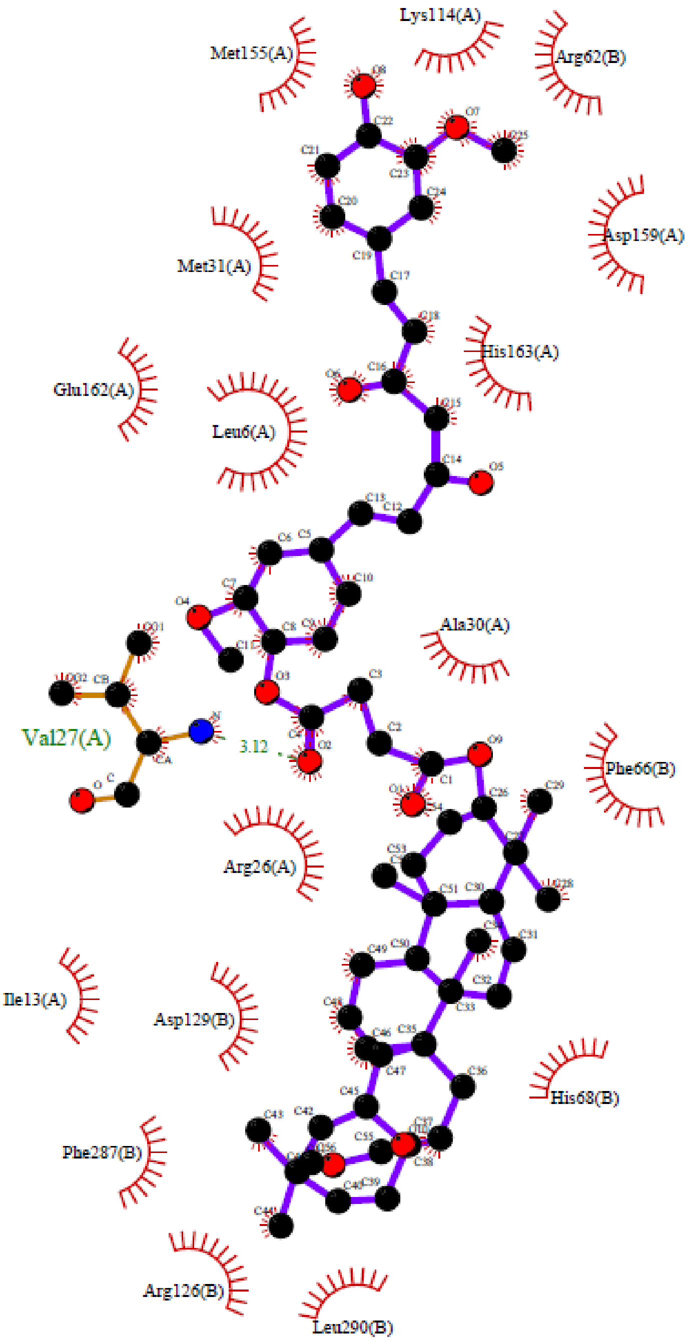

Figure S13. The first poses of the docked ligands to compound **KS3**; hydrogen atoms are omitted; 2w96.pdb protein (LigPlot+ v.2.2 software).

KS4

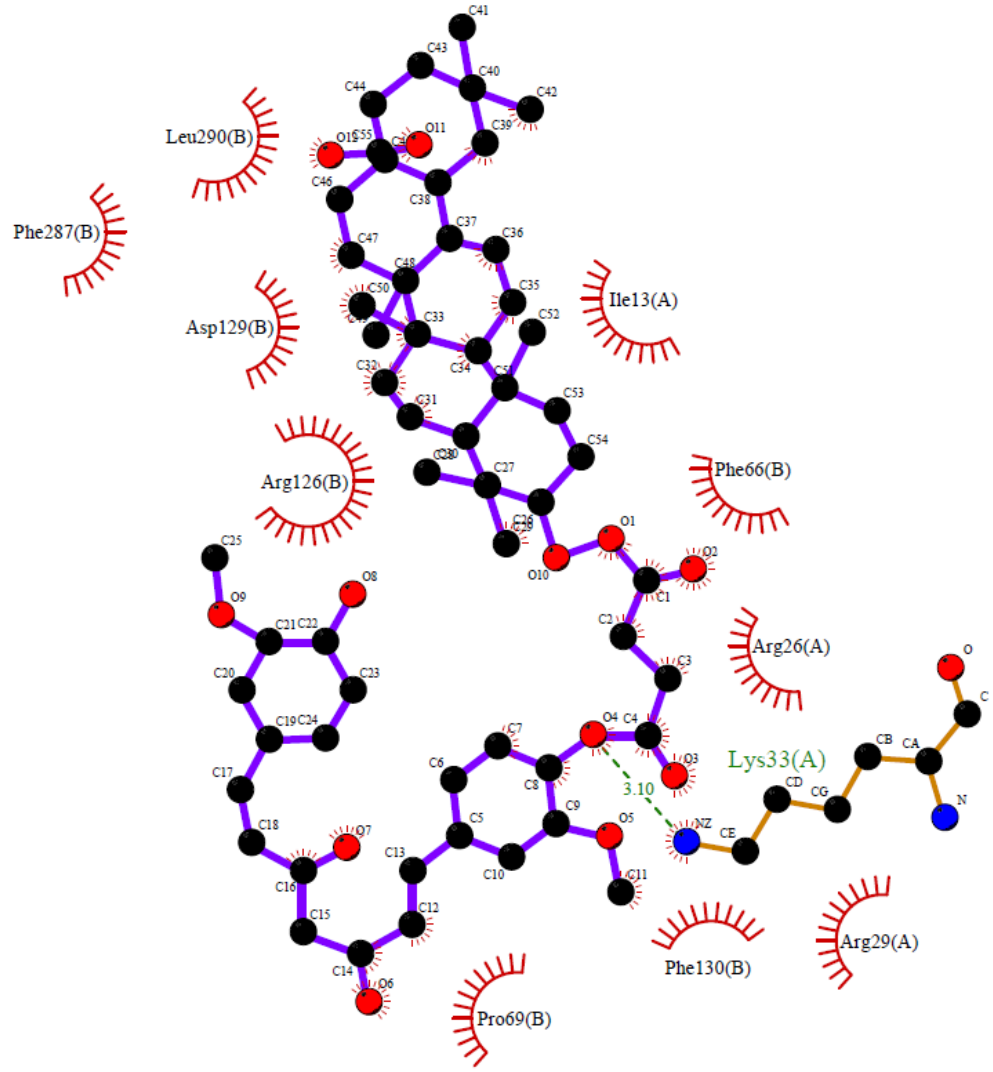

Figure S14 The first poses of the docked ligands to compound **KS4**; hydrogen atoms are omitted; 2w96.pdb protein (LigPlot+ v.2.2 software).

KS5

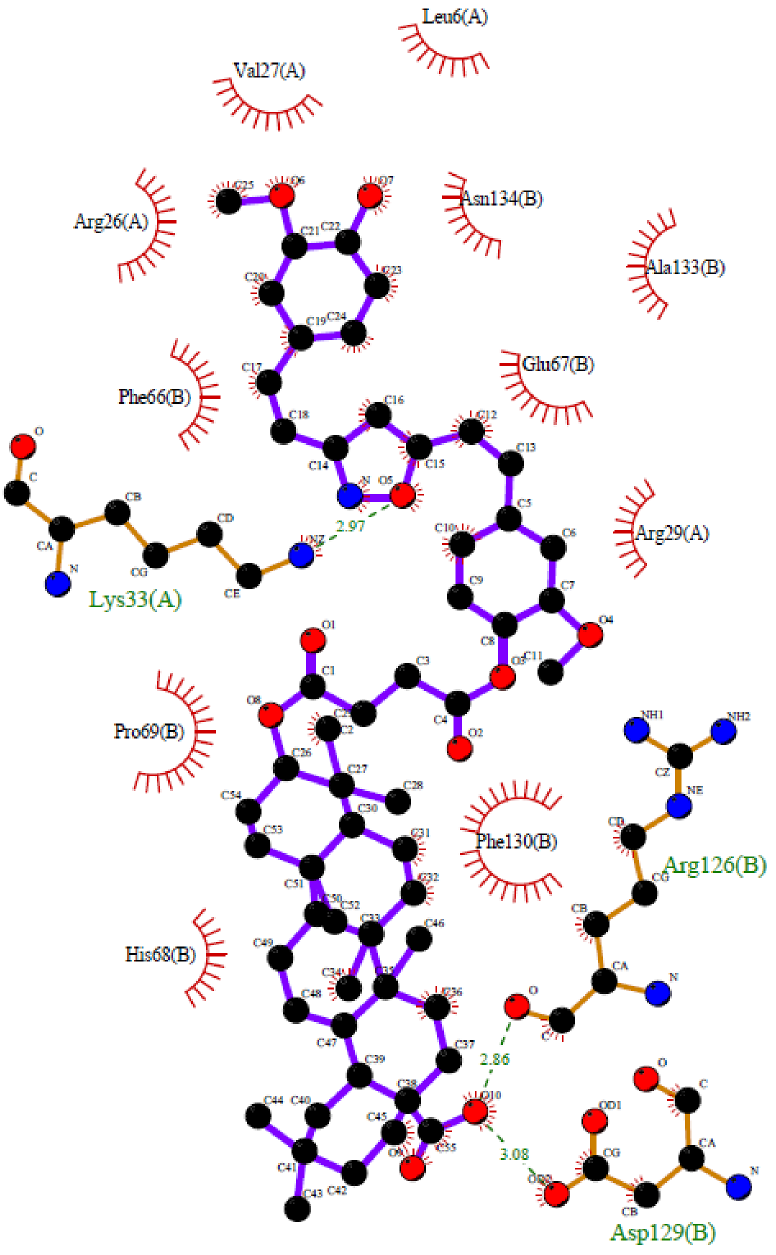

Figure S15 The first poses of the docked ligands to compound **KS5**; hydrogen atoms are omitted; 2w96.pdb protein (LigPlot+ v.2.2 software).

KS6

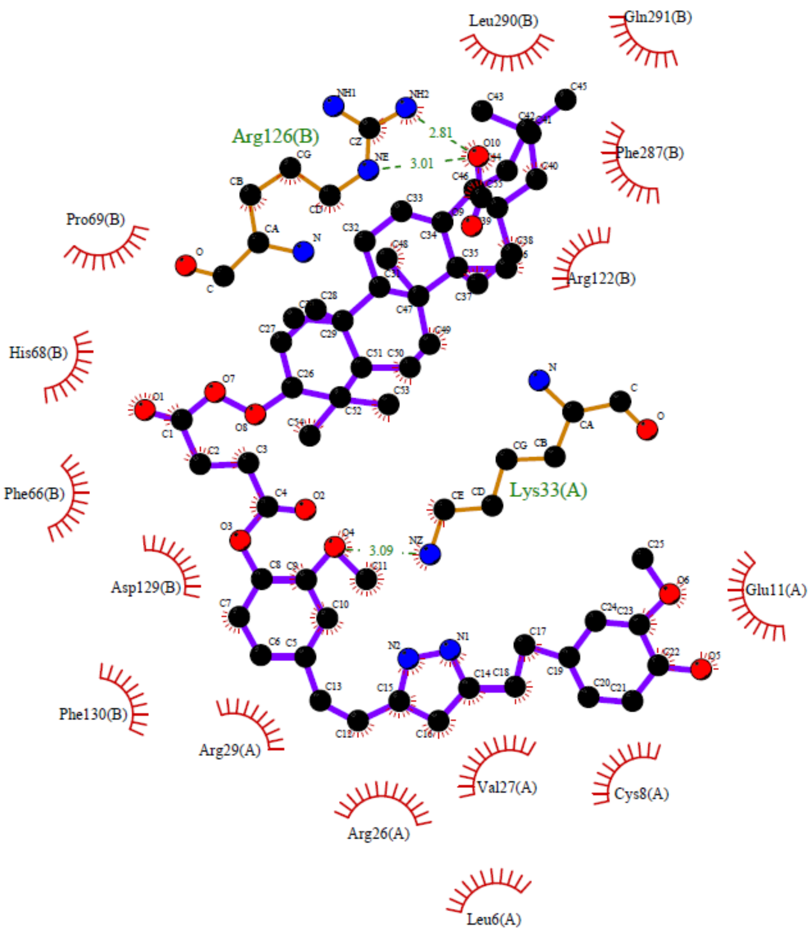

Figure S16 The first poses of the docked ligands to compound **KS6**; hydrogen atoms are omitted; 2w96.pdb protein (LigPlot+ v.2.2 software).

KS7

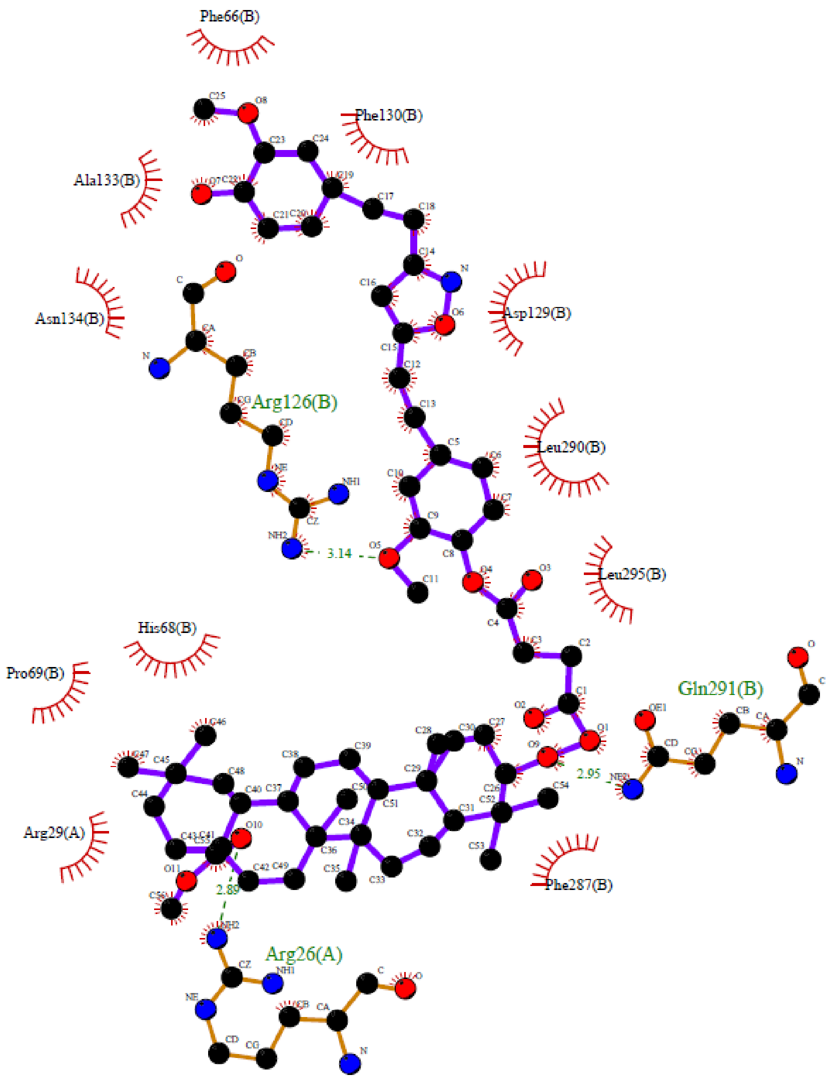

Figure S17 The first poses of the docked ligands to compound **KS7**; hydrogen atoms are omitted; 2w96.pdb protein (LigPlot+ v.2.2 software).

KS8

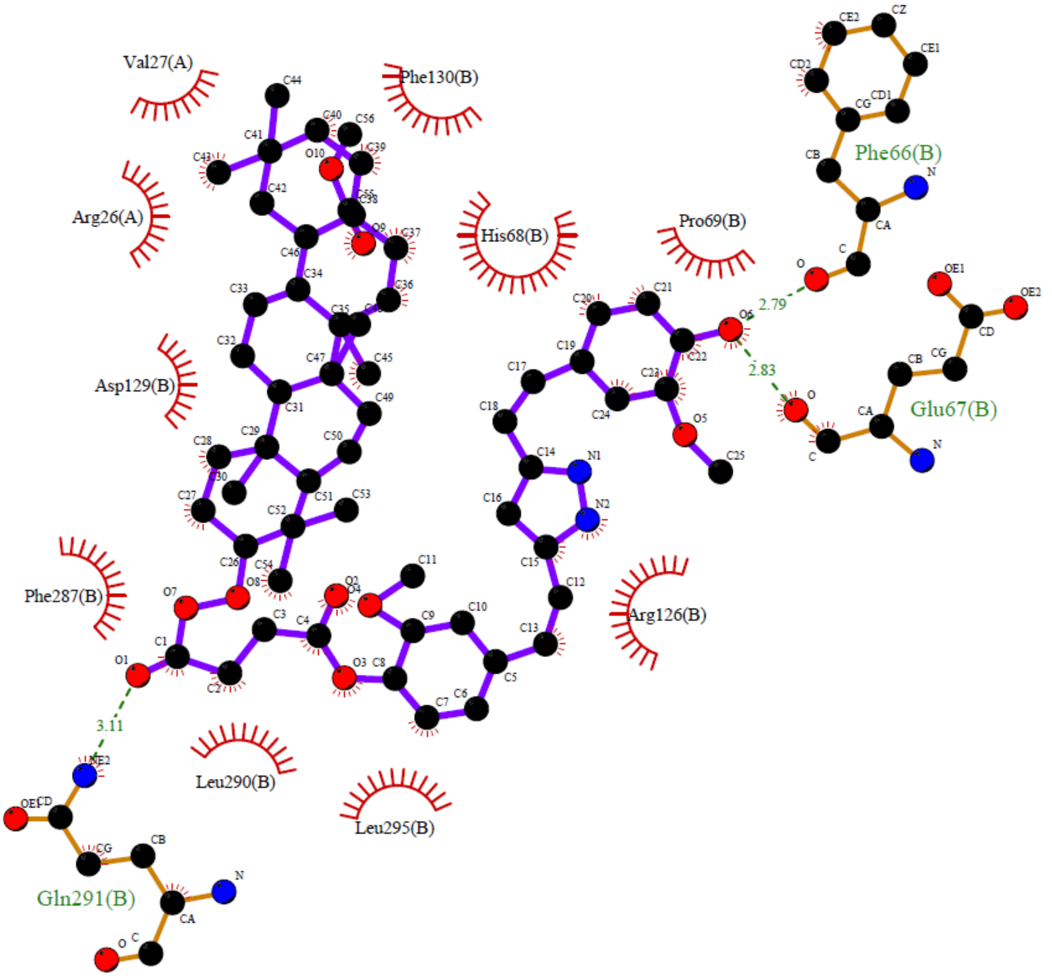

Figure S18 The first poses of the docked ligands to compound **KS8**; hydrogen atoms are omitted; 2w96.pdb protein (LigPlot+ v.2.2 software).

KS1

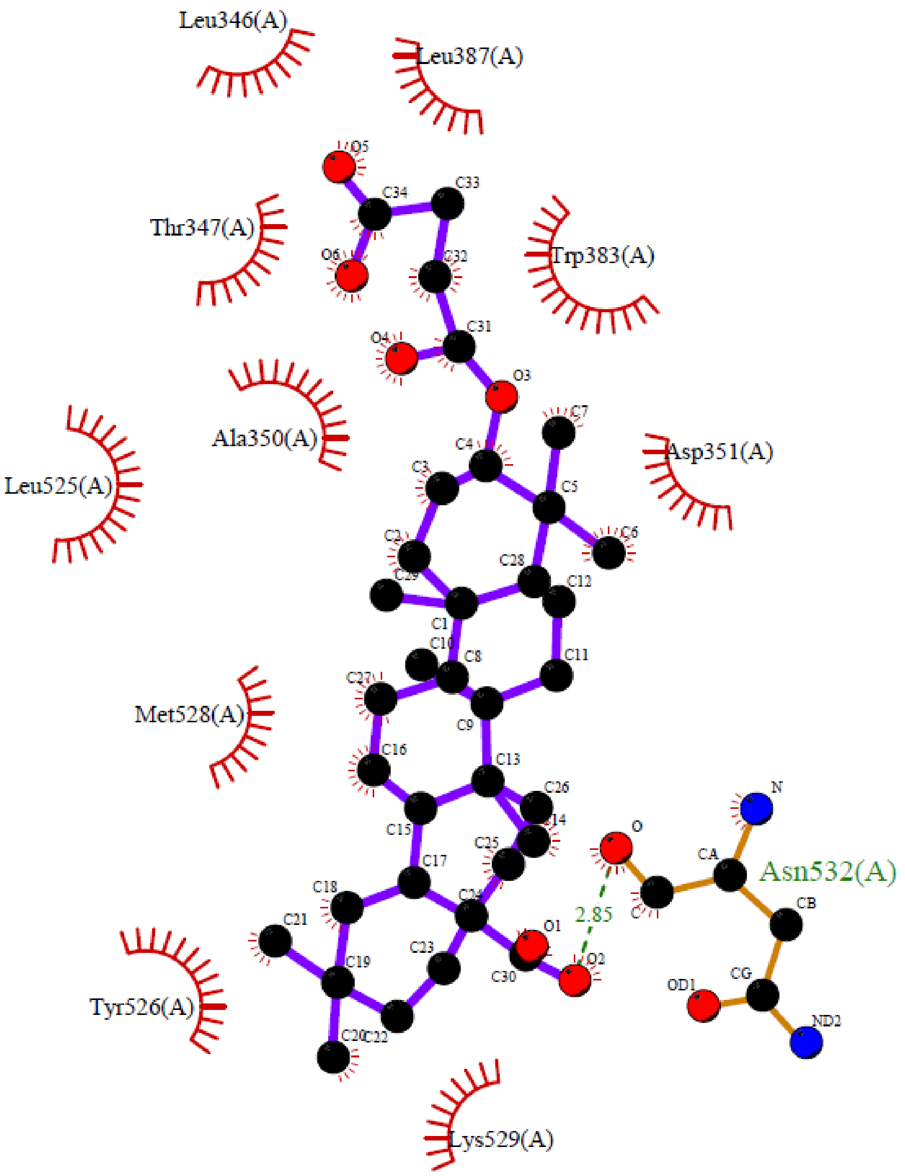

Figure S19 The first poses of the docked ligands to compound **KS1**; hydrogen atoms are omitted; 3dt3.pdb protein (LigPlot+ v.2.2 software).

KS2_keto

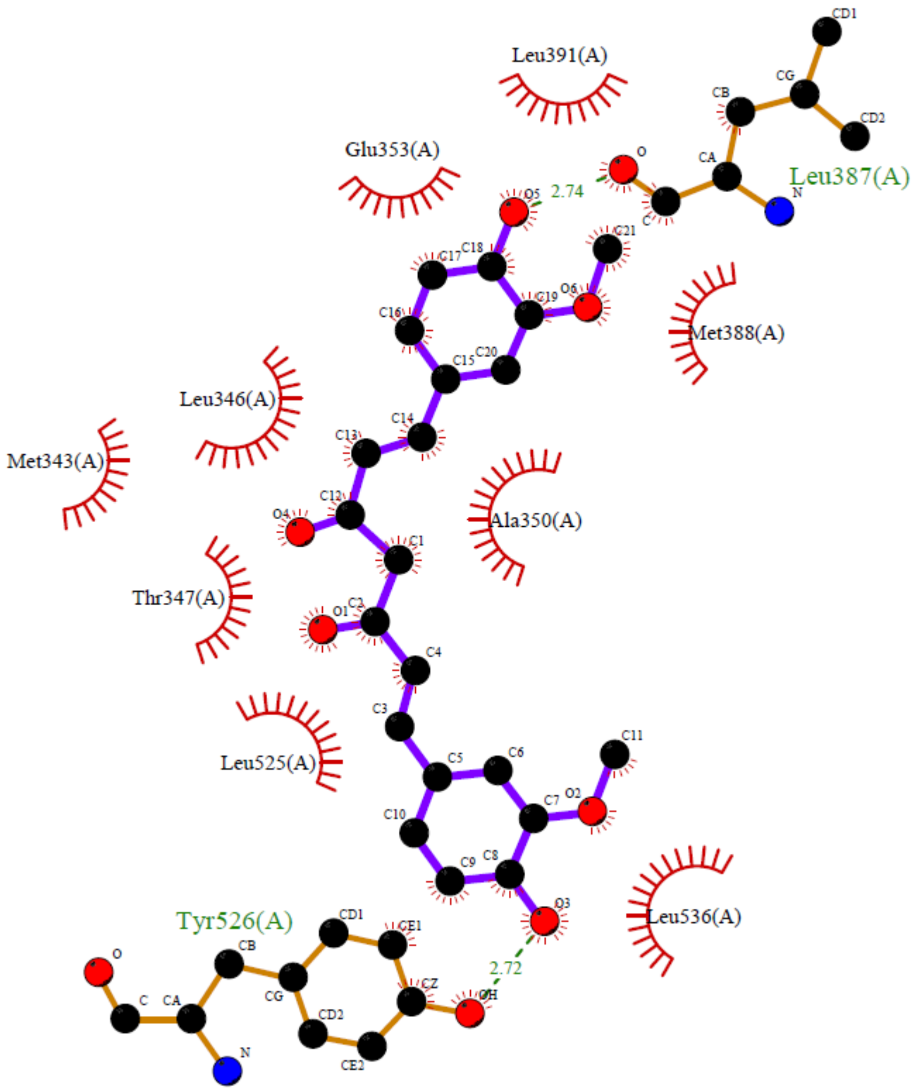

Figure S20a The first poses of the docked ligands to compound **KS2_keto**; hydrogen atoms are omitted; 3dt3.pdb protein (LigPlot+ v.2.2 software).

KS2_enol

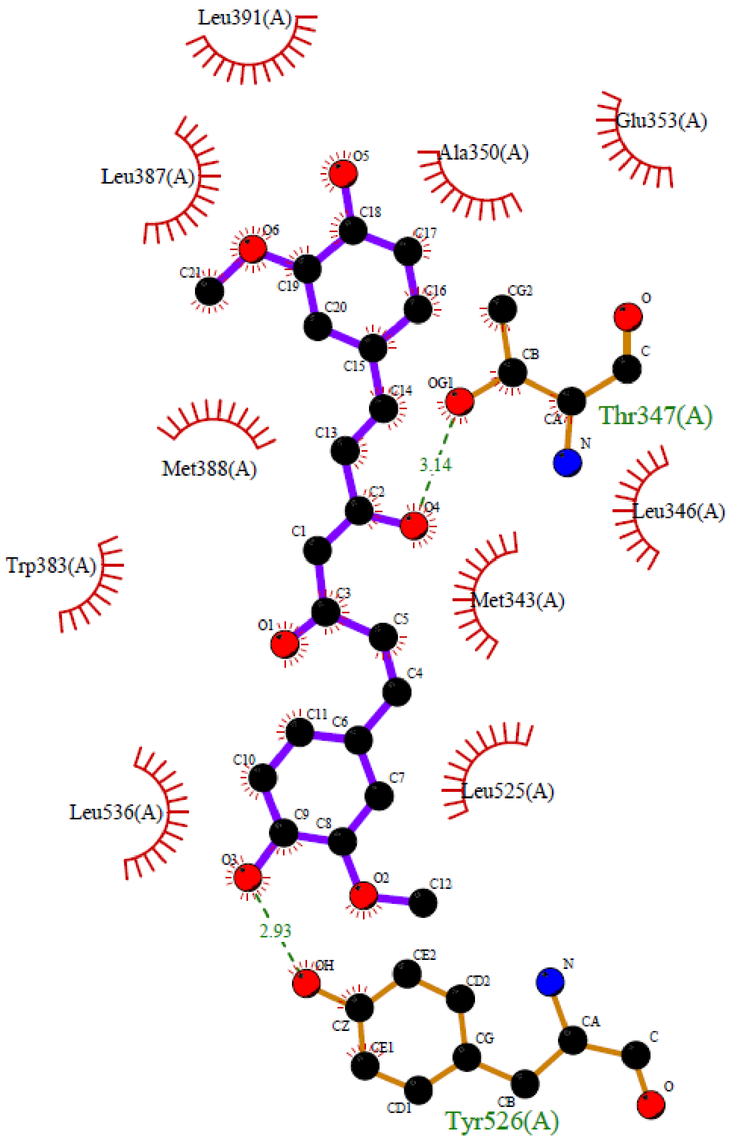

Figure S20b The first poses of the docked ligands to compound **KS2_enol**; hydrogen atoms are omitted; 3dt3.pdb protein (LigPlot+ v.2.2 software).

KS3

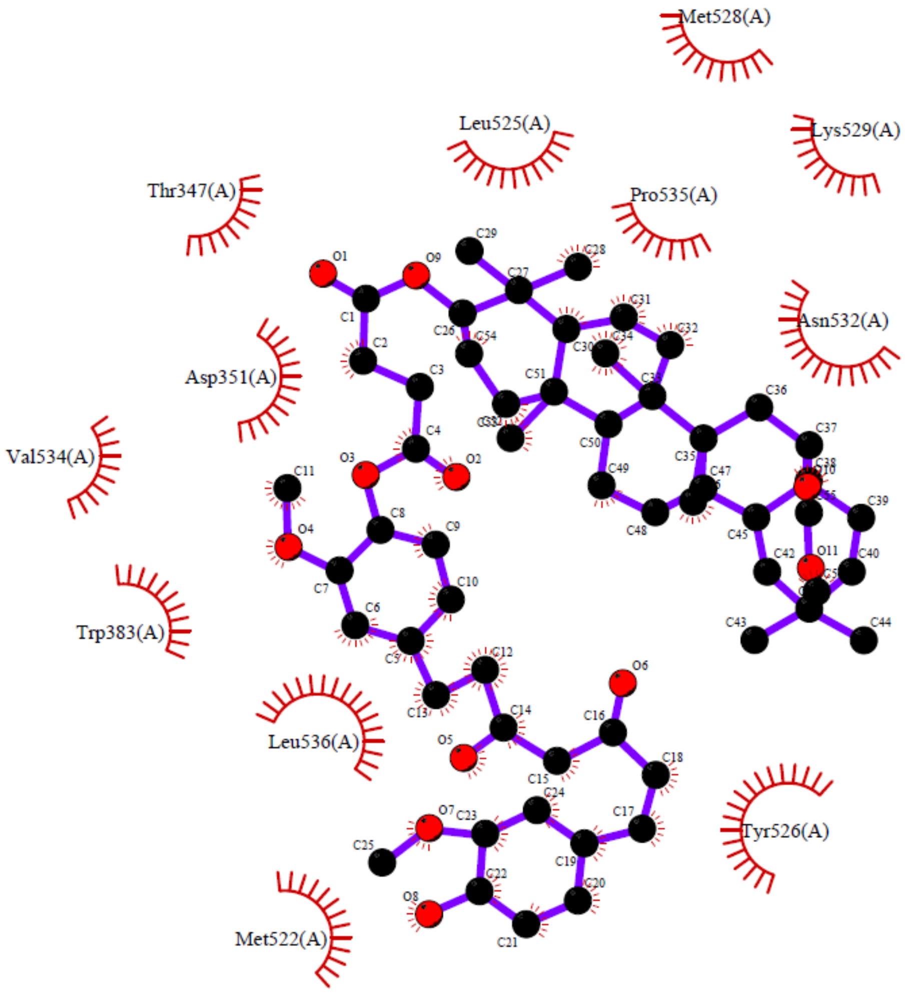

Figure S21 The first poses of the docked ligands to compound **KS3**; hydrogen atoms are omitted; 3dt3.pdb protein (LigPlot+ v.2.2 software).

KS4

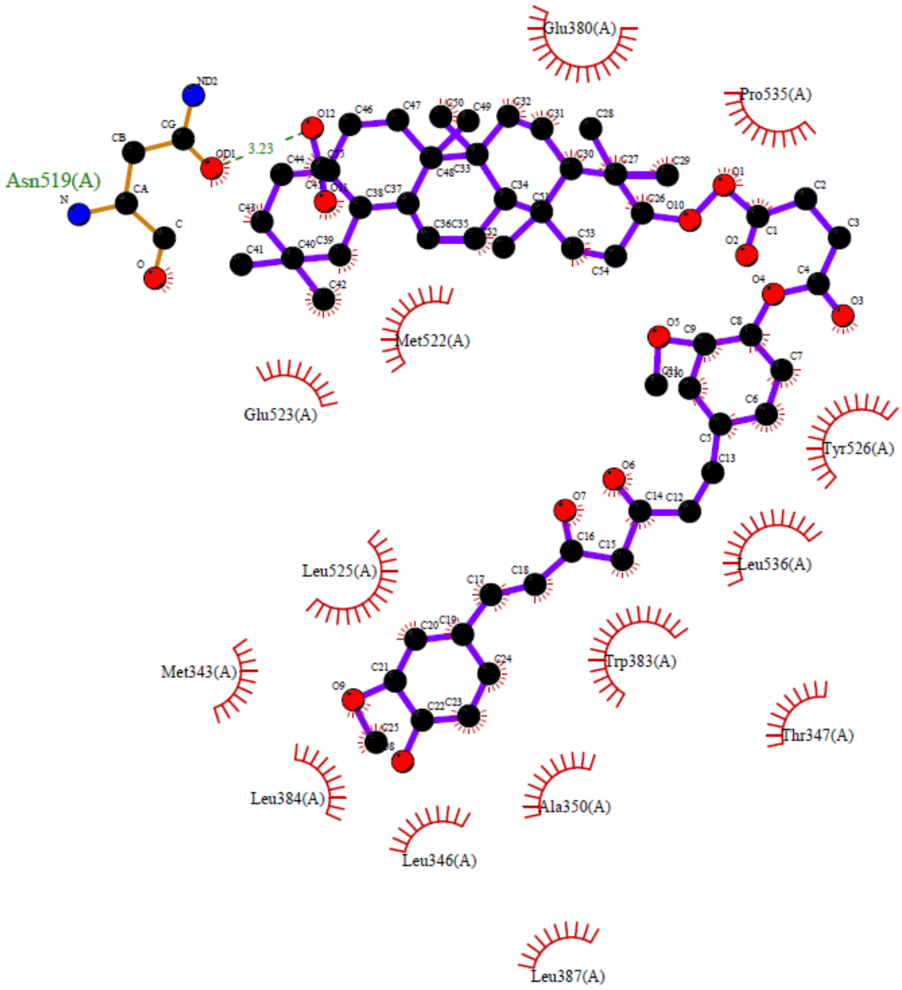

Figure S22 The first poses of the docked ligands to compound **KS4**; hydrogen atoms are omitted; 3dt3.pdb protein (LigPlot+ v.2.2 software).

KS5

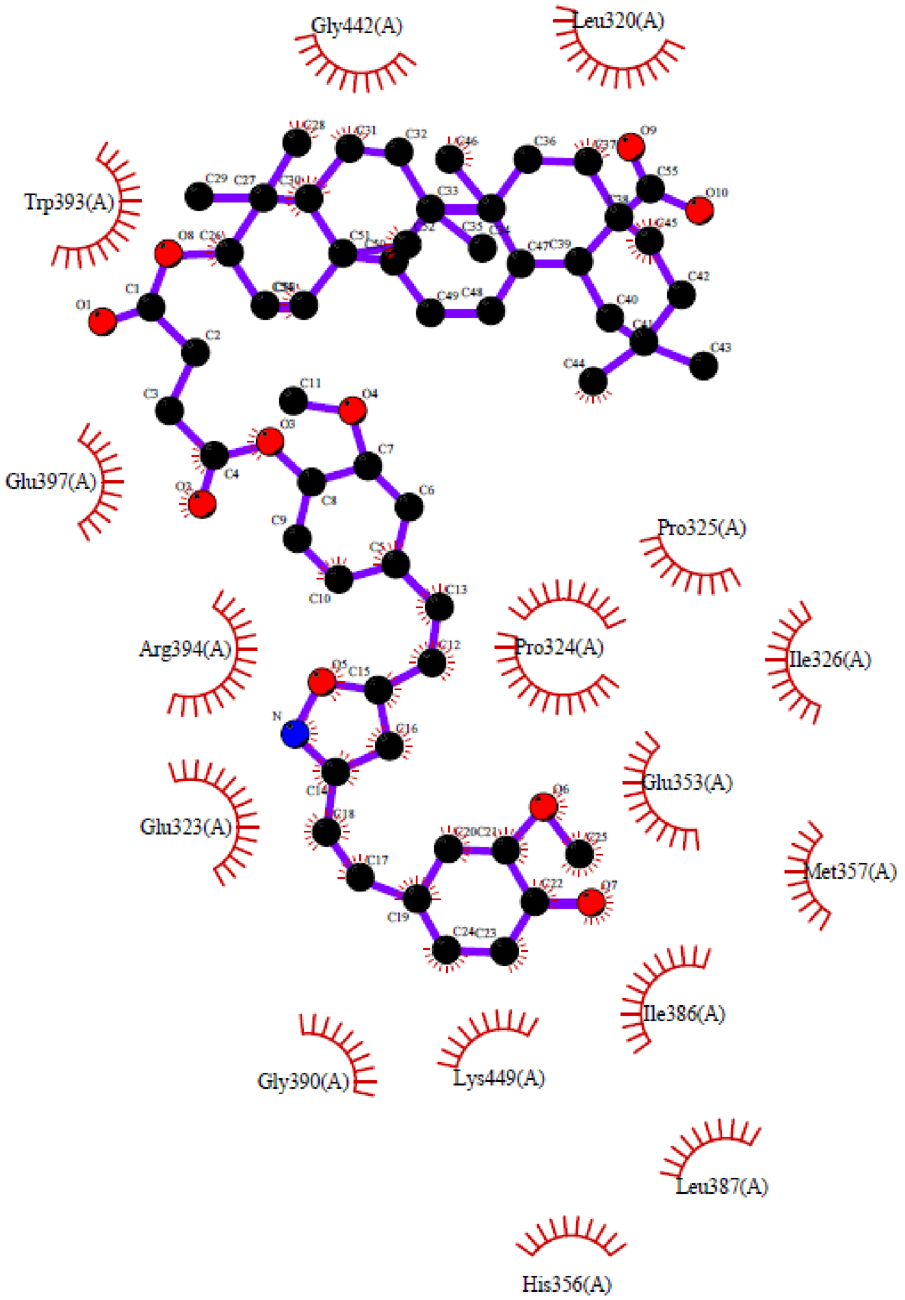

Figure S23 The first poses of the docked ligands to compound **KS5**; hydrogen atoms are omitted; 3dt3.pdb protein (LigPlot+ v.2.2 software).

KS6

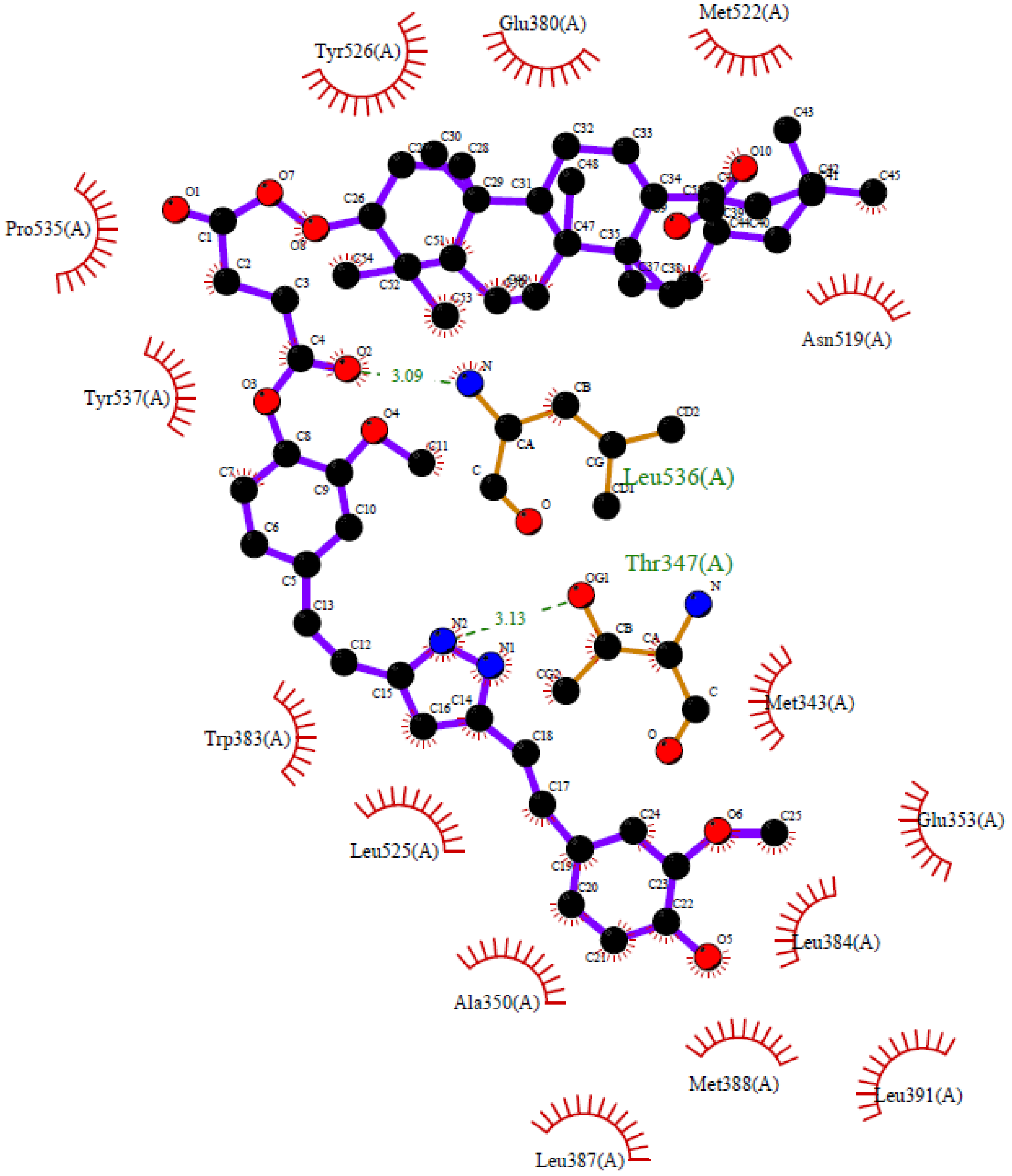

Figure S24 The first poses of the docked ligands to compound **KS6**; hydrogen atoms are omitted; 3dt3.pdb protein (LigPlot+ v.2.2 software).

KS7

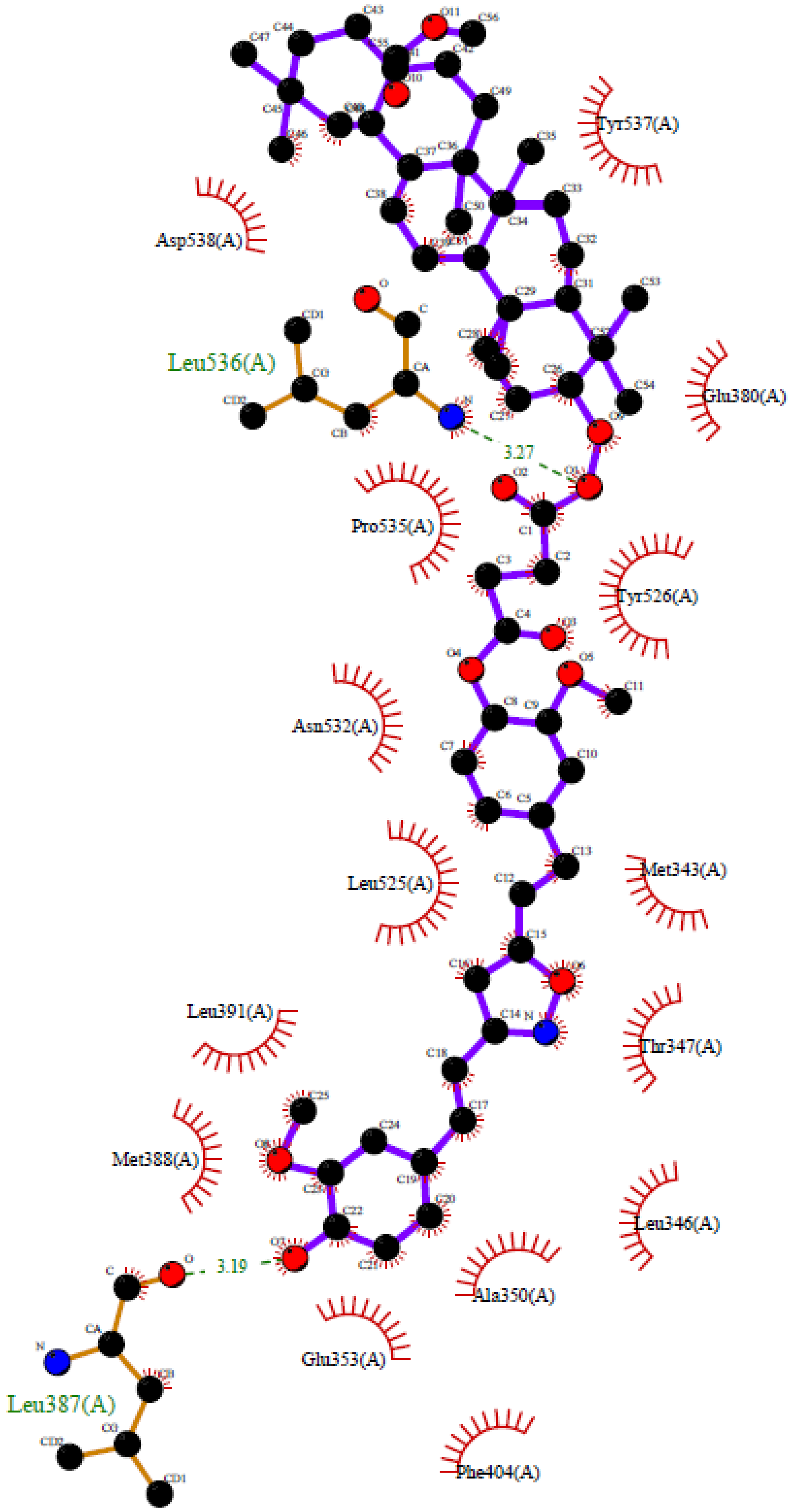

Figure S25 The first poses of the docked ligands to compound **KS7**; hydrogen atoms are omitted; 3dt3.pdb protein (LigPlot+ v.2.2 software).

KS8

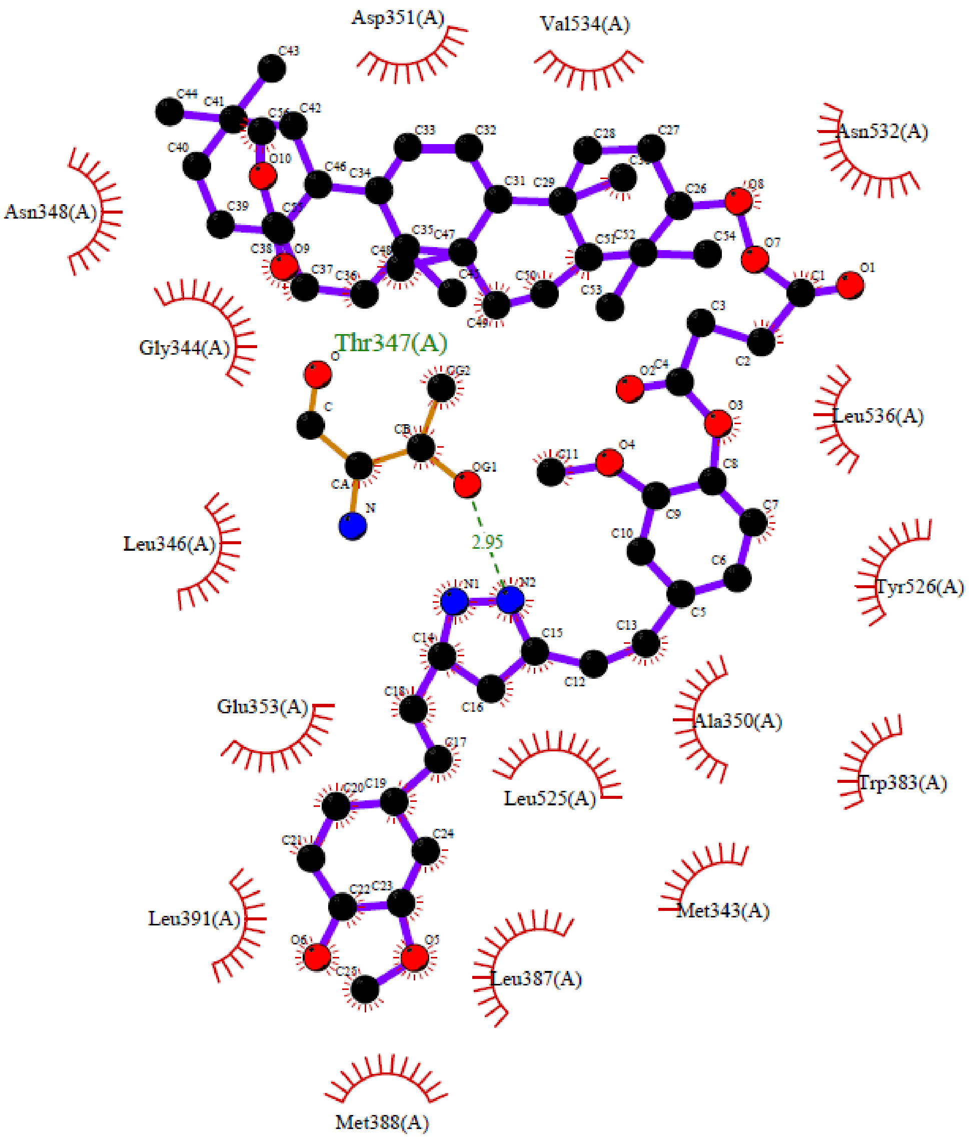

Figure S26 The first poses of the docked ligands to compound **KS8**; hydrogen atoms are omitted; 3dt3.pdb protein (LigPlot+ v.2.2 software).

KS1

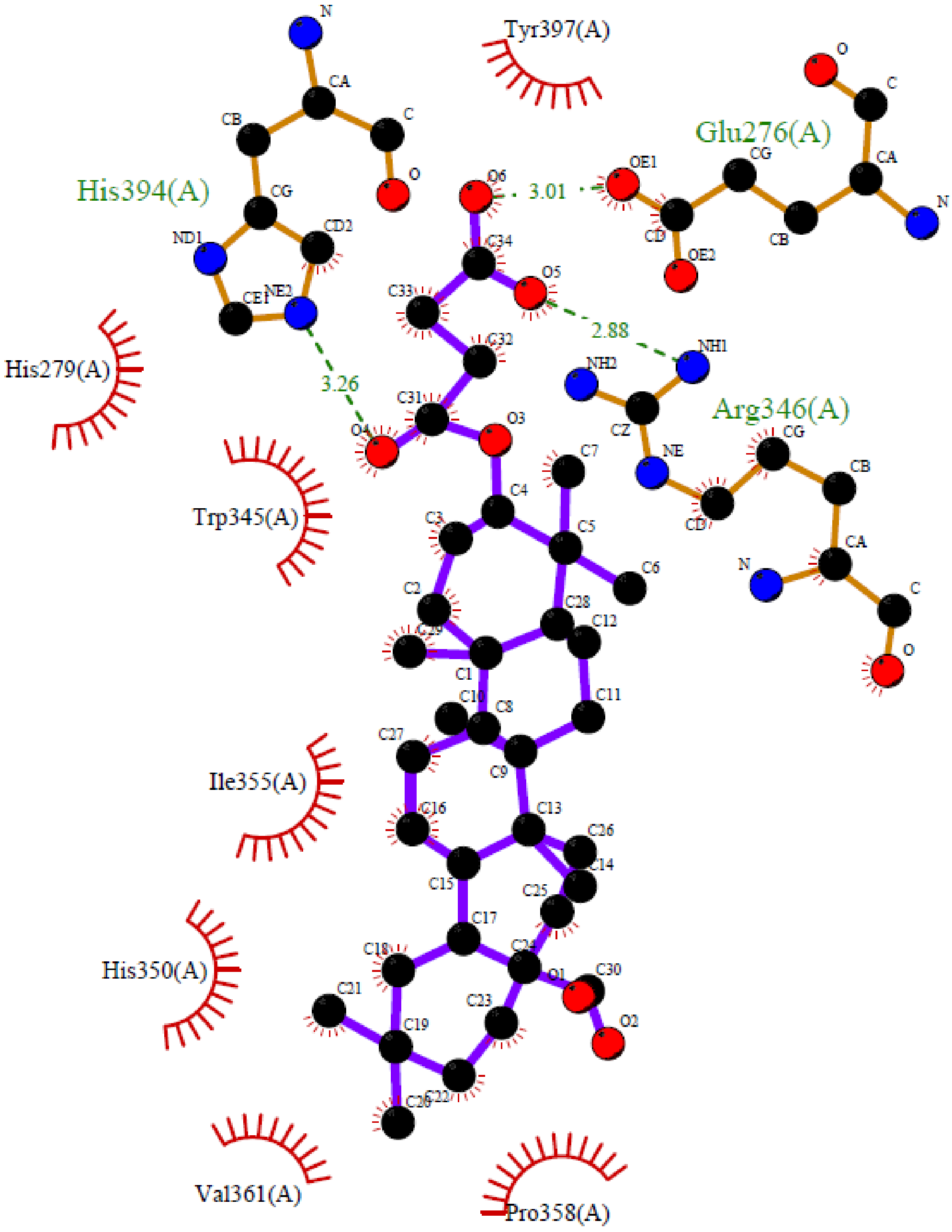

Figure S27 The first poses of the docked ligands to compound **KS1**; hydrogen atoms are omitted; 1l2j.pdb protein (LigPlot+ v.2.2 software).

KS2_keto

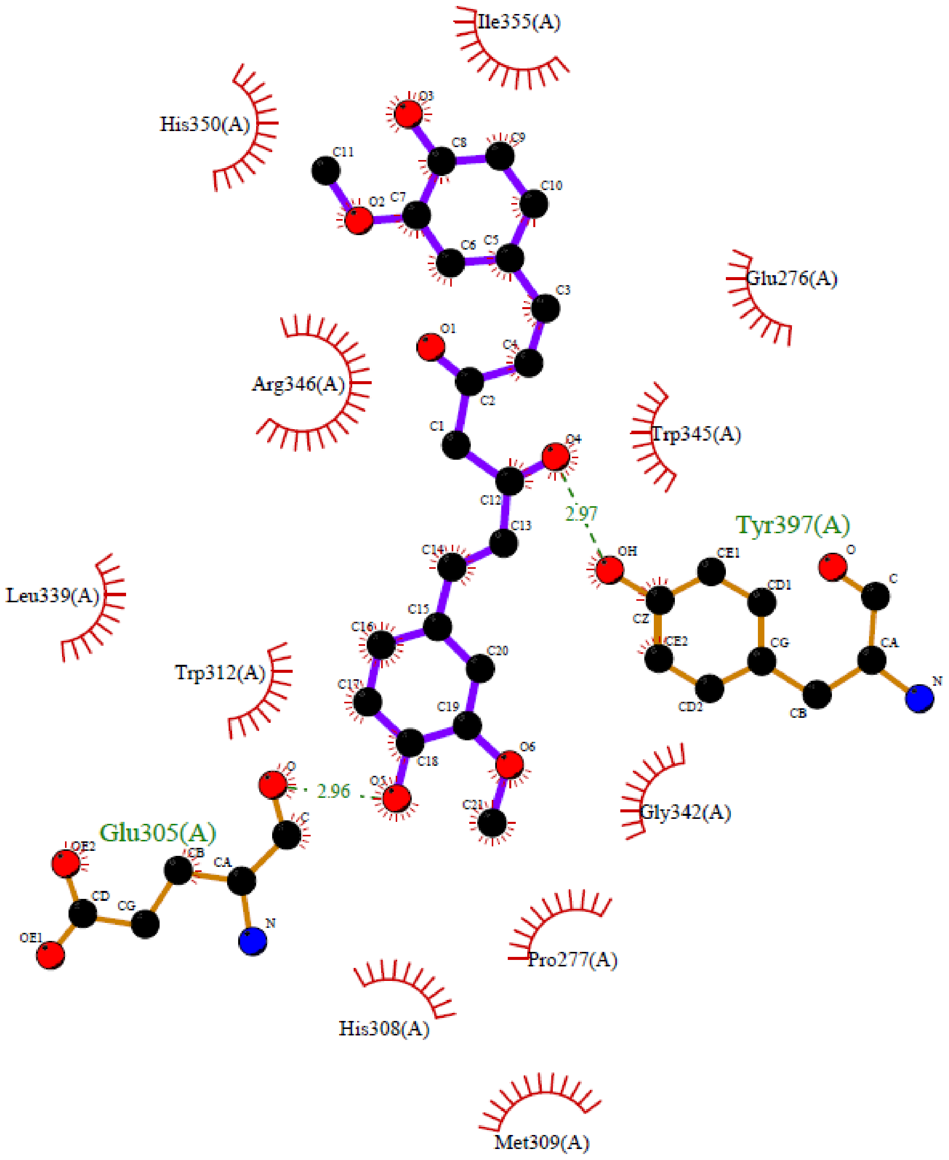

Figure S28 The first poses of the docked ligands to compound **KS2_keto**; hydrogen atoms are omitted; 1l2j.pdb protein (LigPlot+ v.2.2 software).

KS2_enol

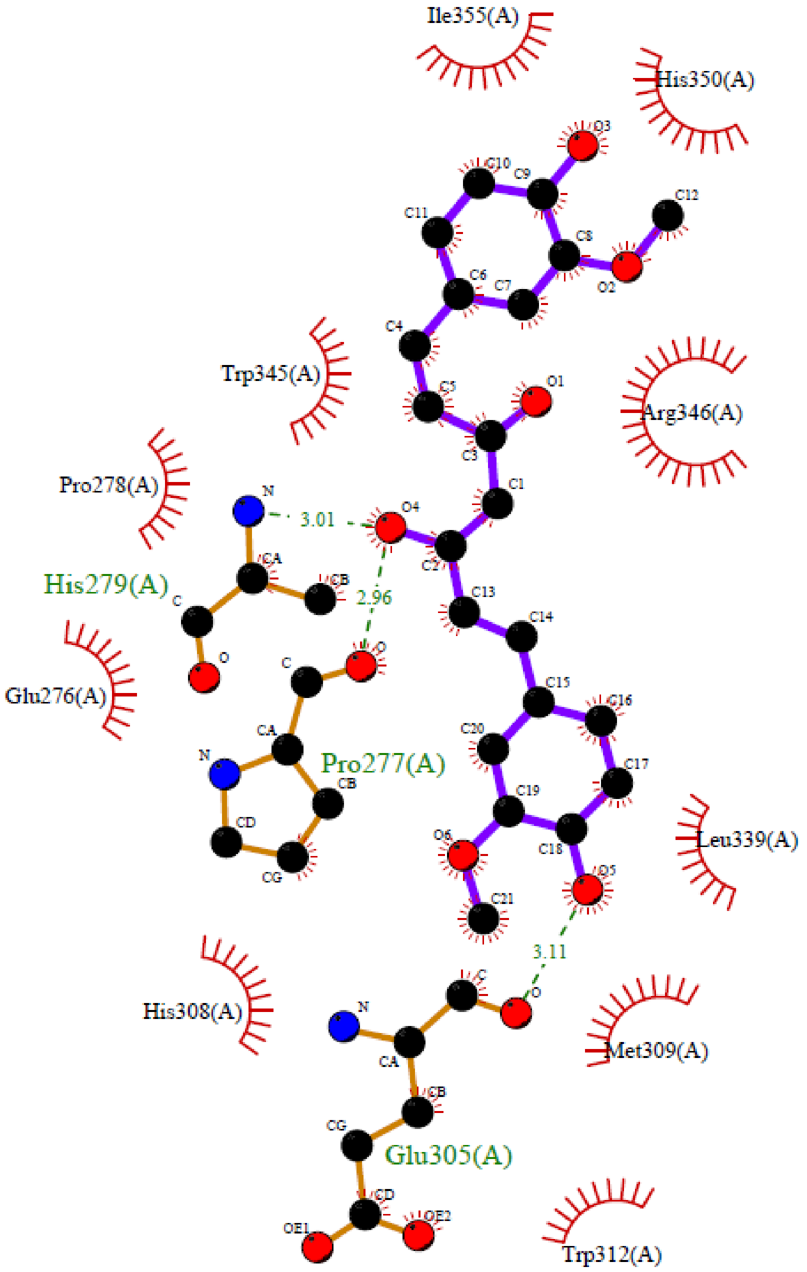

Figure S29 The first poses of the docked ligands to compound **KS2_enol**; hydrogen atoms are omitted; 1l2j.pdb protein (LigPlot+ v.2.2 software).

KS3

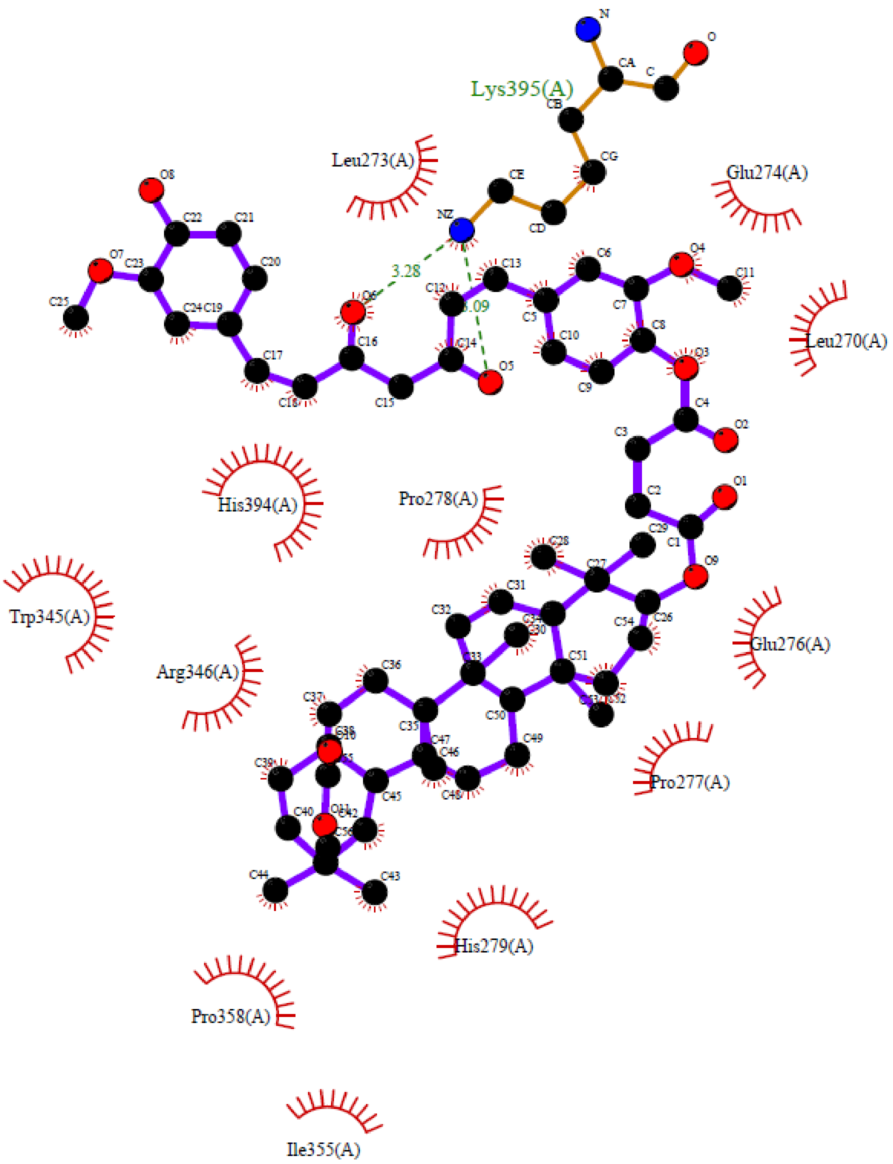

Figure S30 The first poses of the docked ligands to compound **KS3**; hydrogen atoms are omitted; 1l2j.pdb protein (LigPlot+ v.2.2 software).

KS4

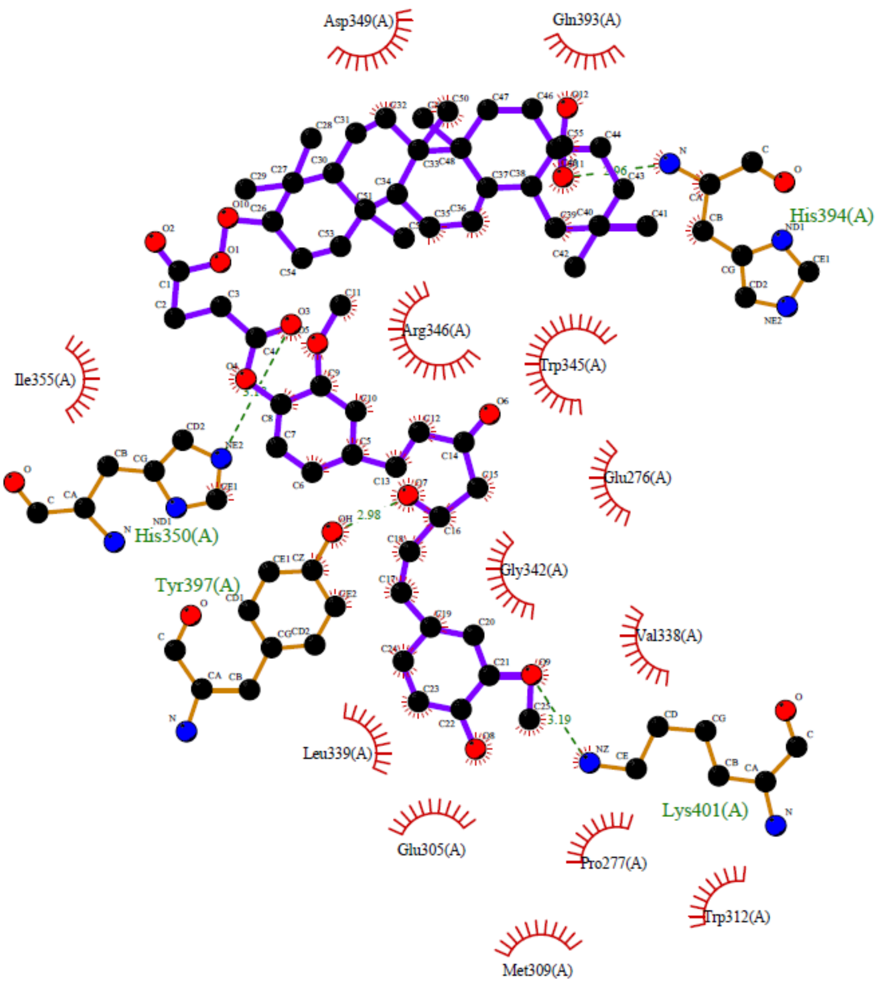

Figure S31 The first poses of the docked ligands to compound **KS4**; hydrogen atoms are omitted; 1l2j.pdb protein (LigPlot+ v.2.2 software).

KS5

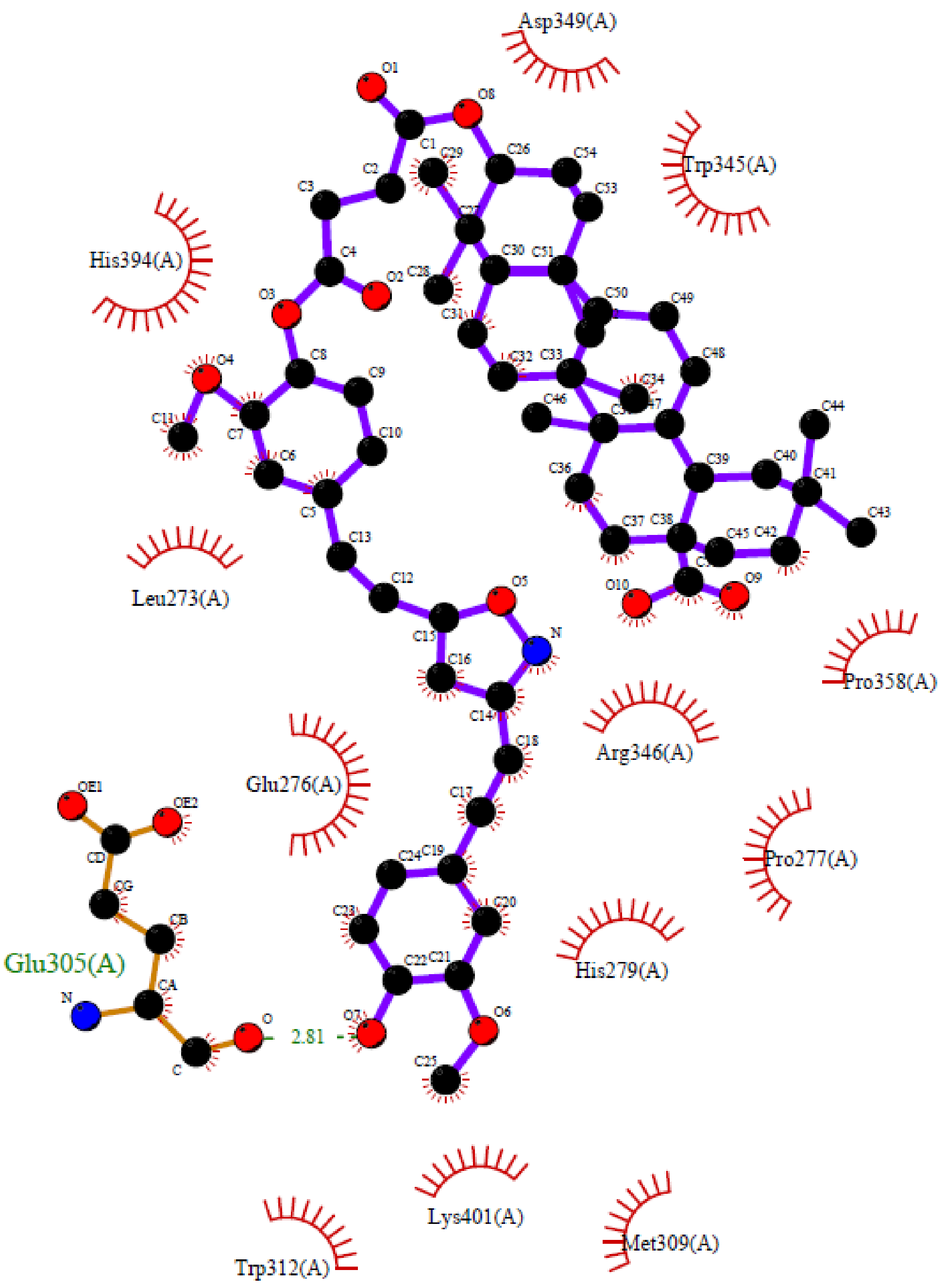

Figure S32 The first poses of the docked ligands to compound **KS6**; hydrogen atoms are omitted; 1l2j.pdb protein (LigPlot+ v.2.2 software).

KS6

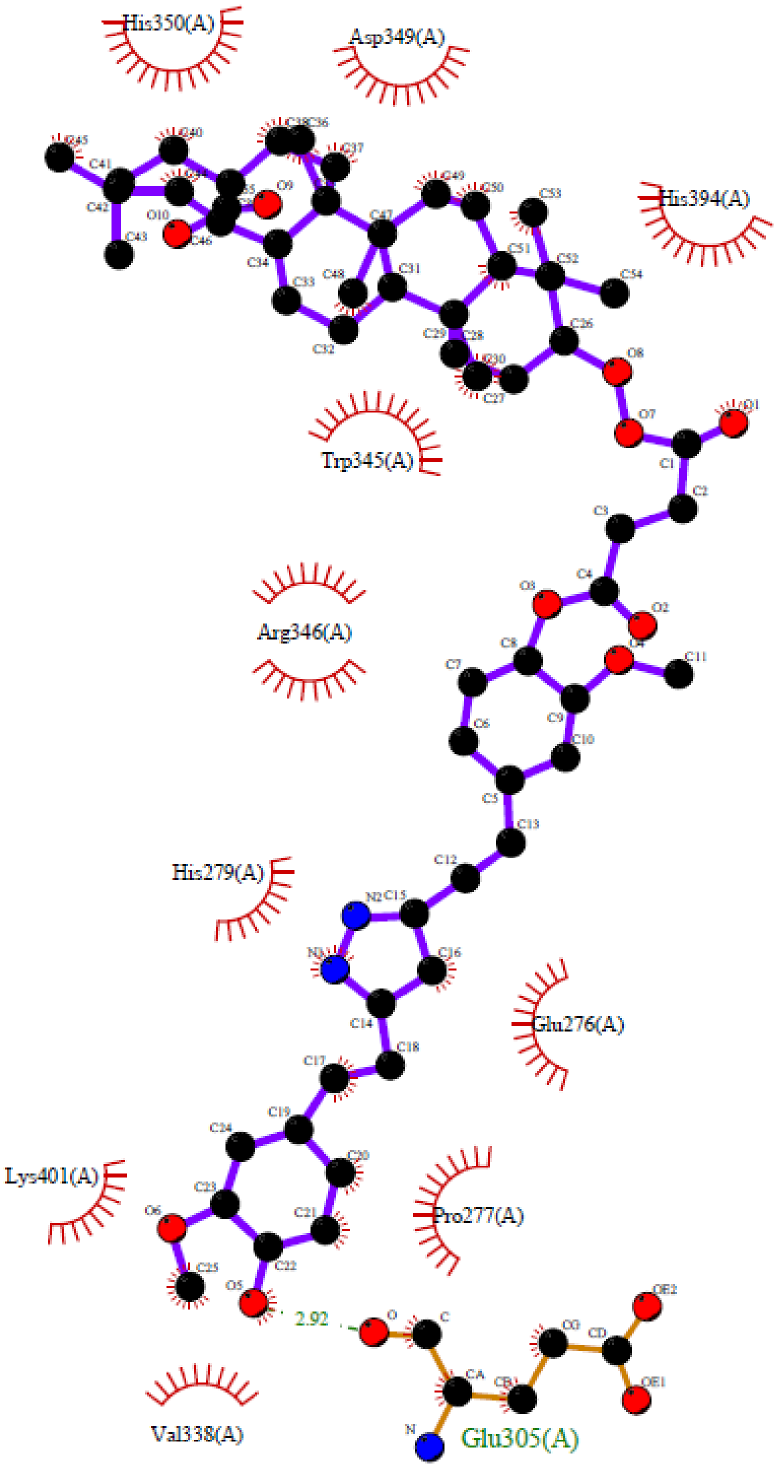

Figure S33 The first poses of the docked ligands to compound **KS6**; hydrogen atoms are omitted; 1l2j.pdb protein (LigPlot+ v.2.2 software).

KS7

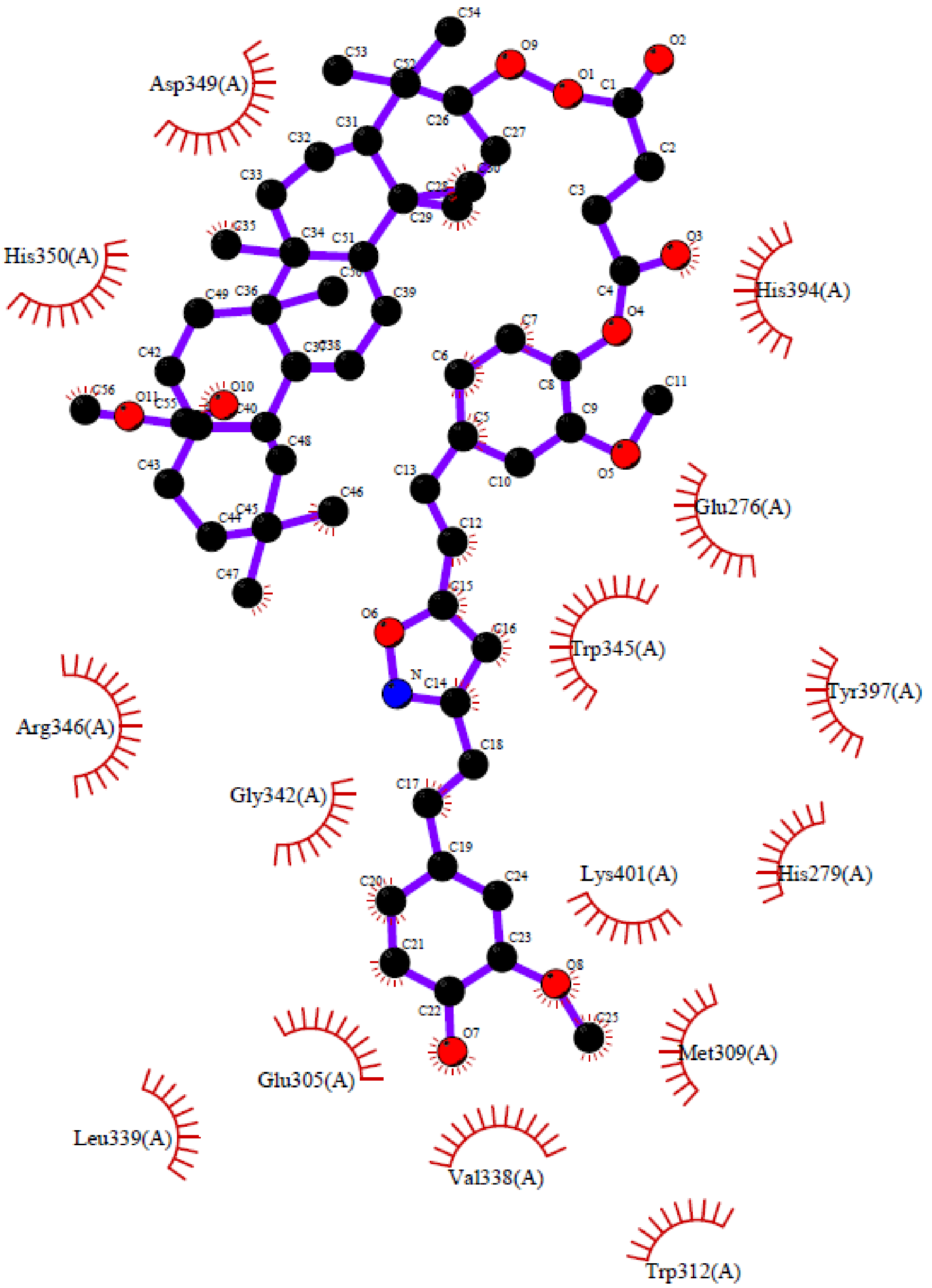

Figure S34 The first poses of the docked ligands to compound **KS7**; hydrogen atoms are omitted; 1l2j.pdb protein (LigPlot+ v.2.2 software).

KS8

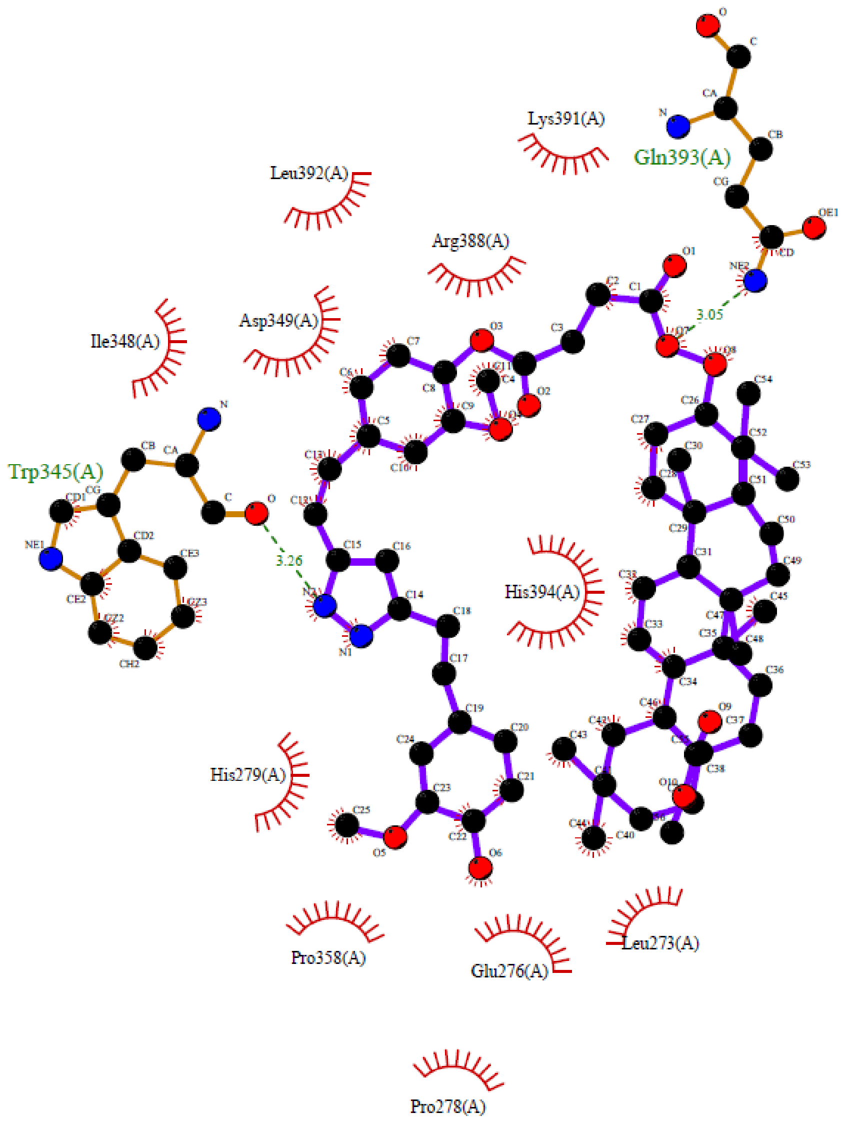

Figure S35 The first poses of the docked ligands to compound **KS8**; hydrogen atoms are omitted; 1l2j.pdb protein (LigPlot+ v.2.2 software).

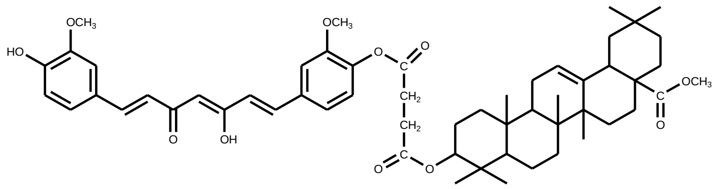

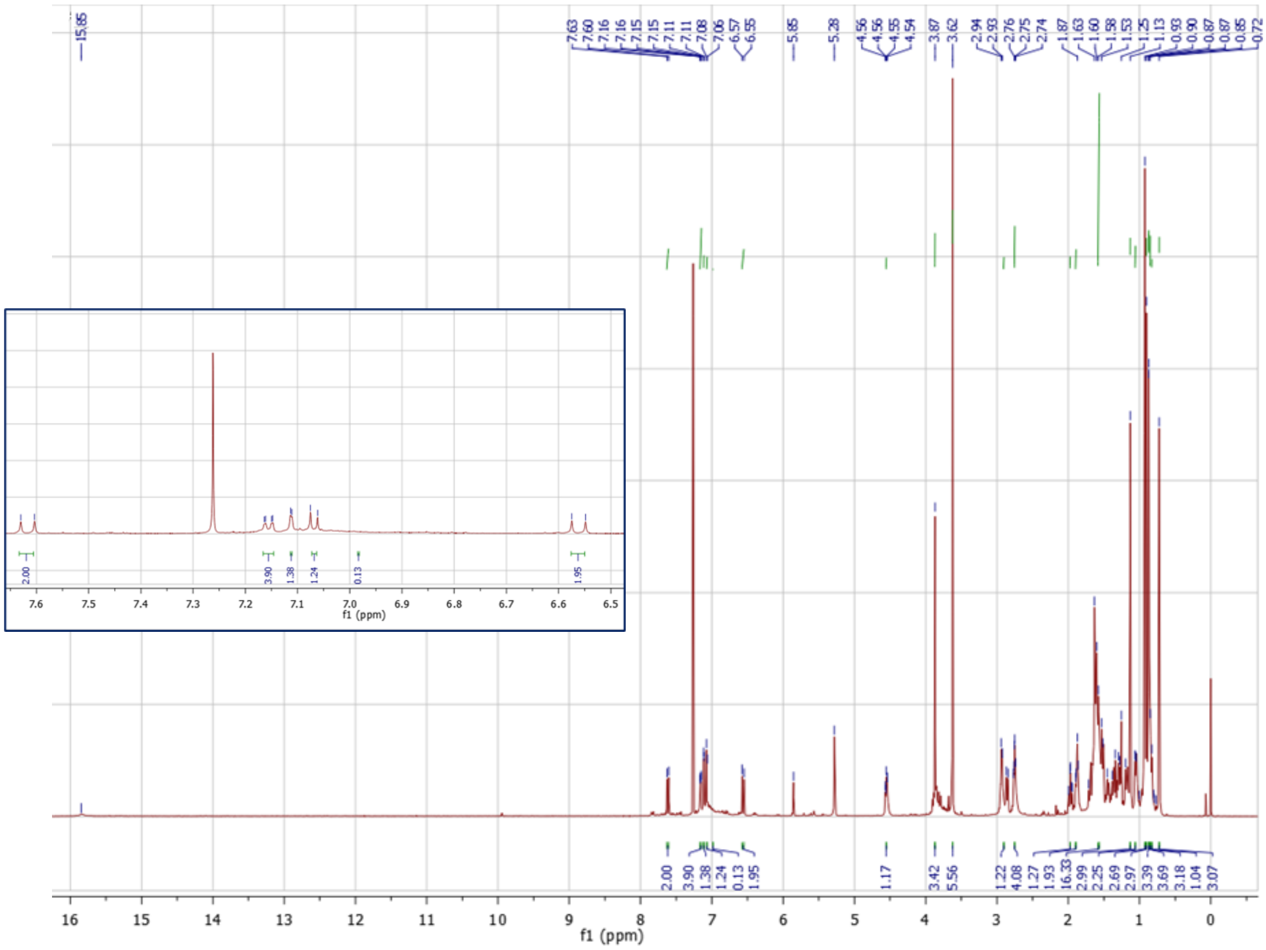

Figure S36 ^{1}H-NMR spectrum of Curcumin mono-oleanoyl hydrogen succinate methyl ester

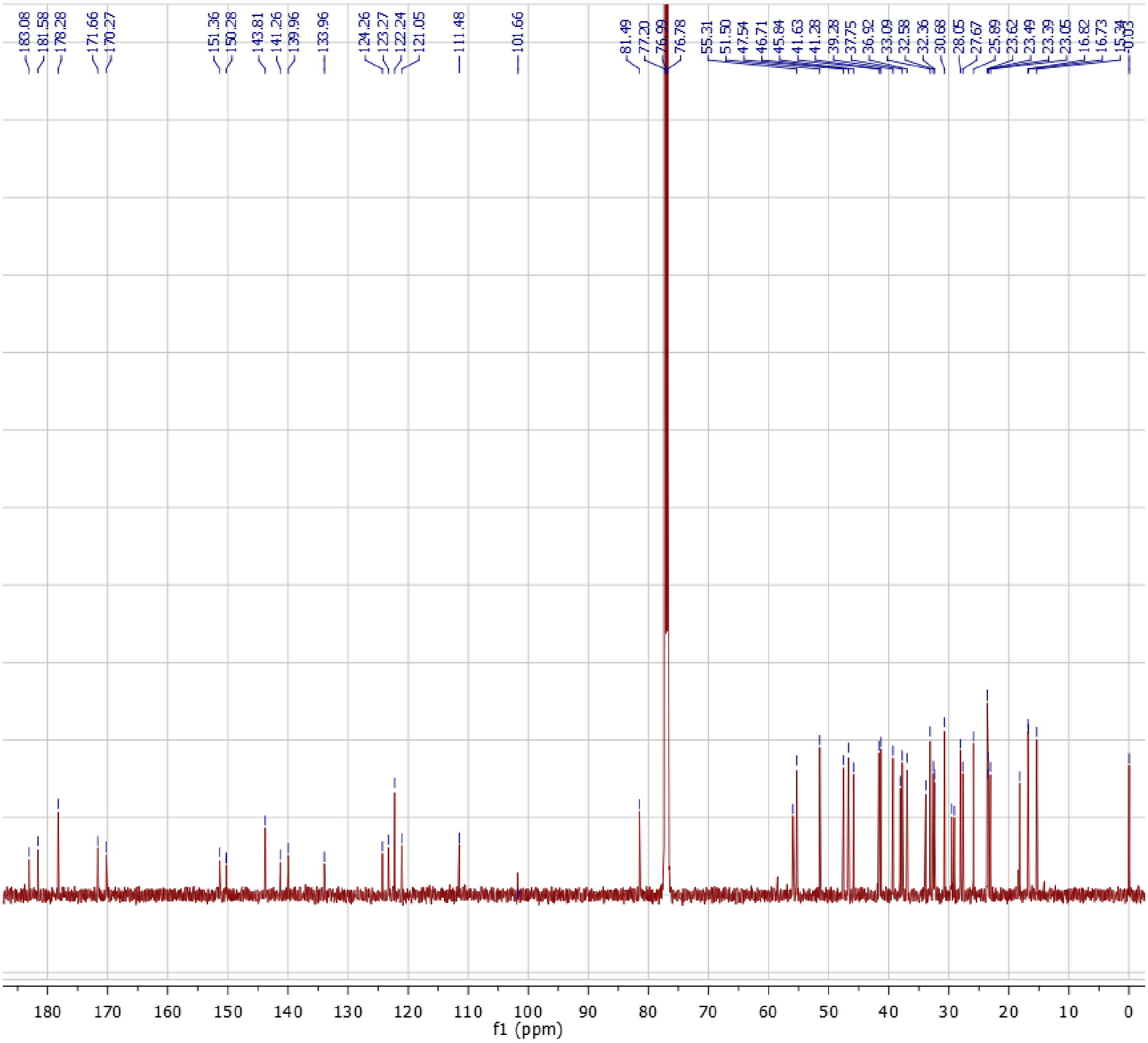

Figure S37 ^{13}C-NMR spectrum of Curcumin mono-oleanoyl hydrogen succinate methyl ester

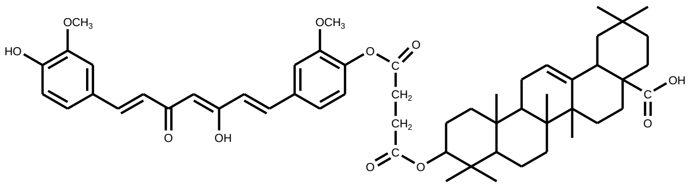

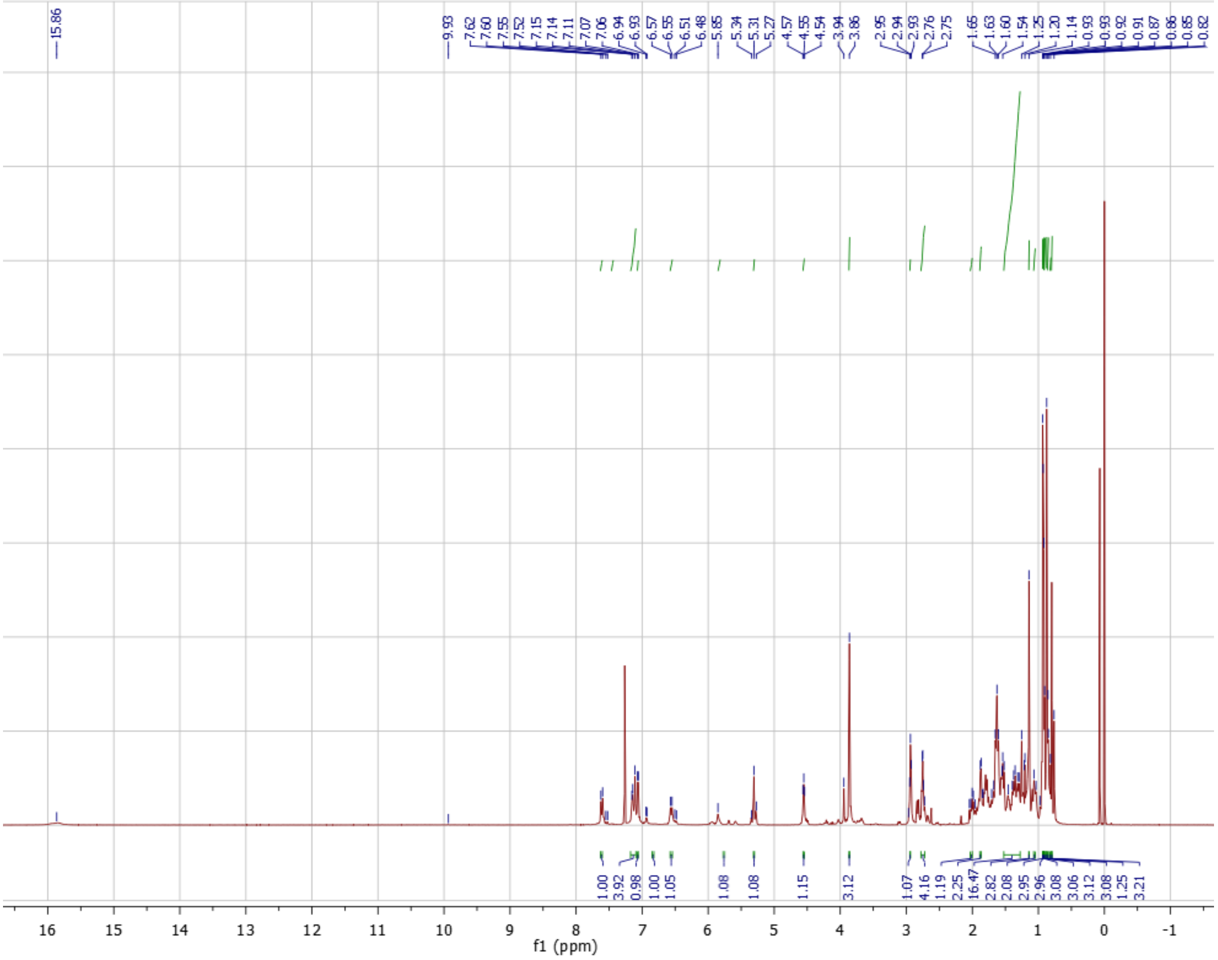

Figure S38 ^{1}H-NMR spectrum of Curcumin mono-oleanoyl hydrogen succinate

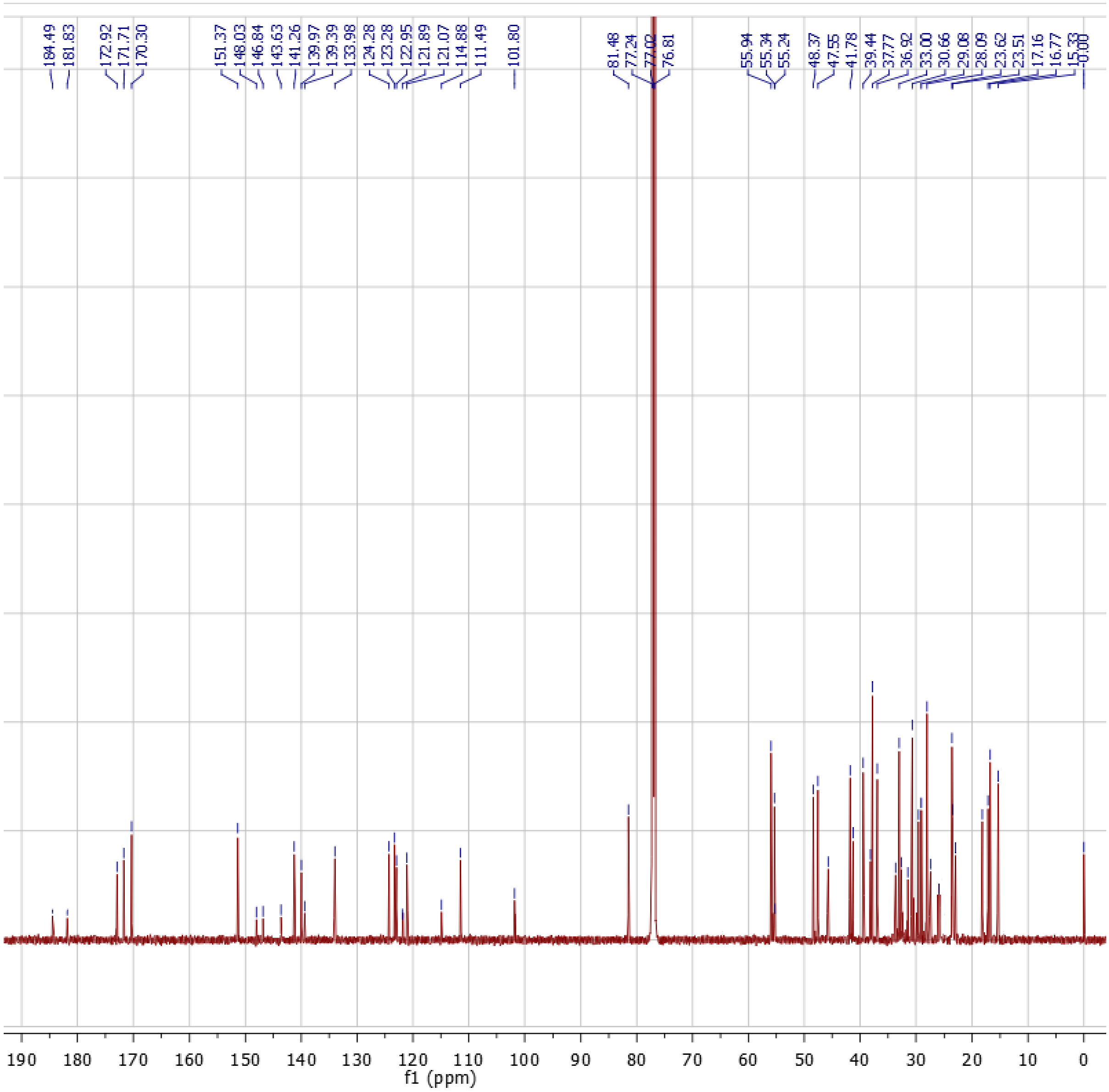

Figure S39 ^{13}C-NMR spectrum of Curcumin mono-oleanoyl hydrogen succinate

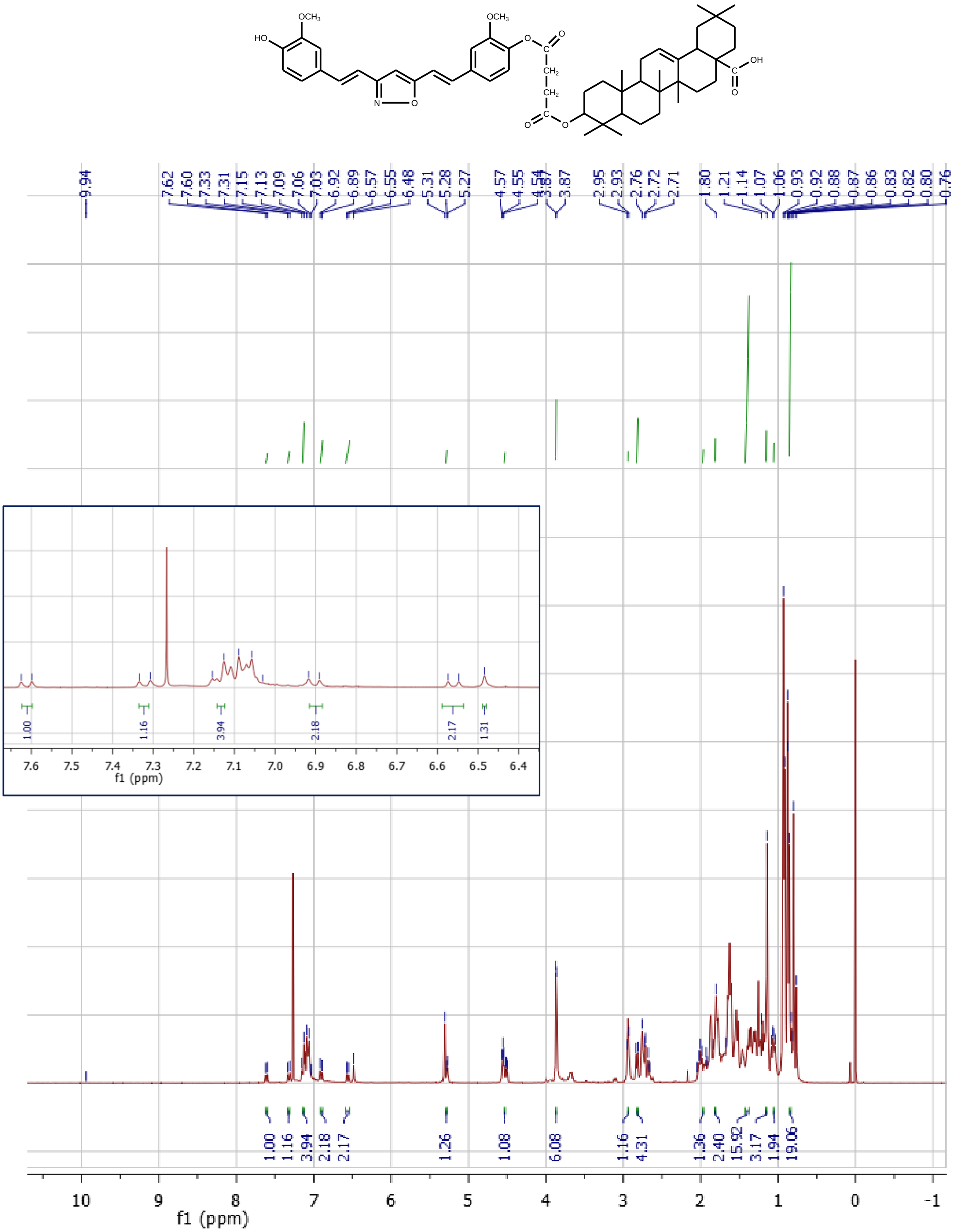

Figure S40 ^{1}H-NMR spectrum of Curcumin isoxazole mono-oleanoyl hydrogen succinate

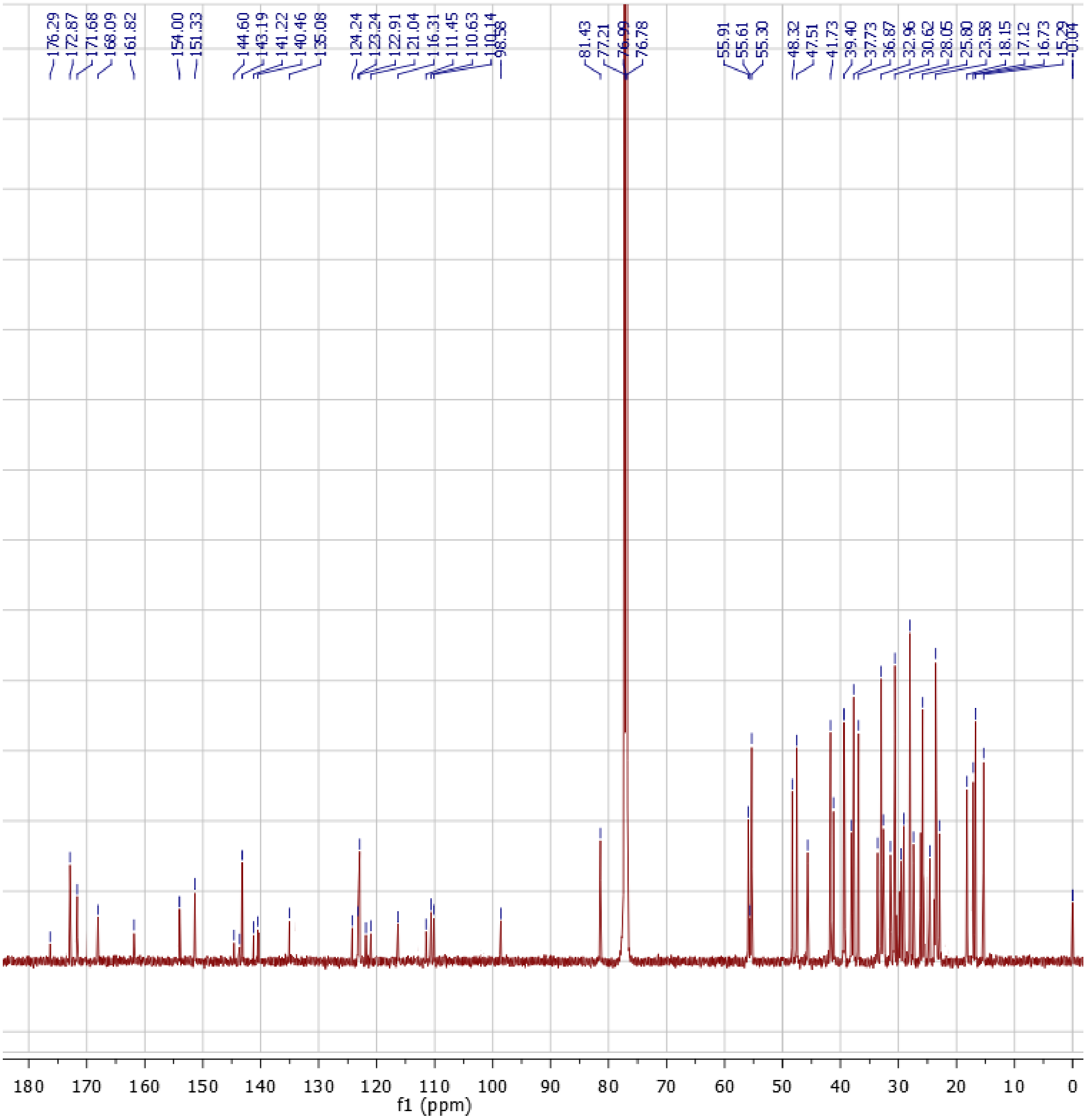

Figure S41 ^{13}C-NMR spectrum of Curcumin isoxazole mono-oleanoyl hydrogen succinate

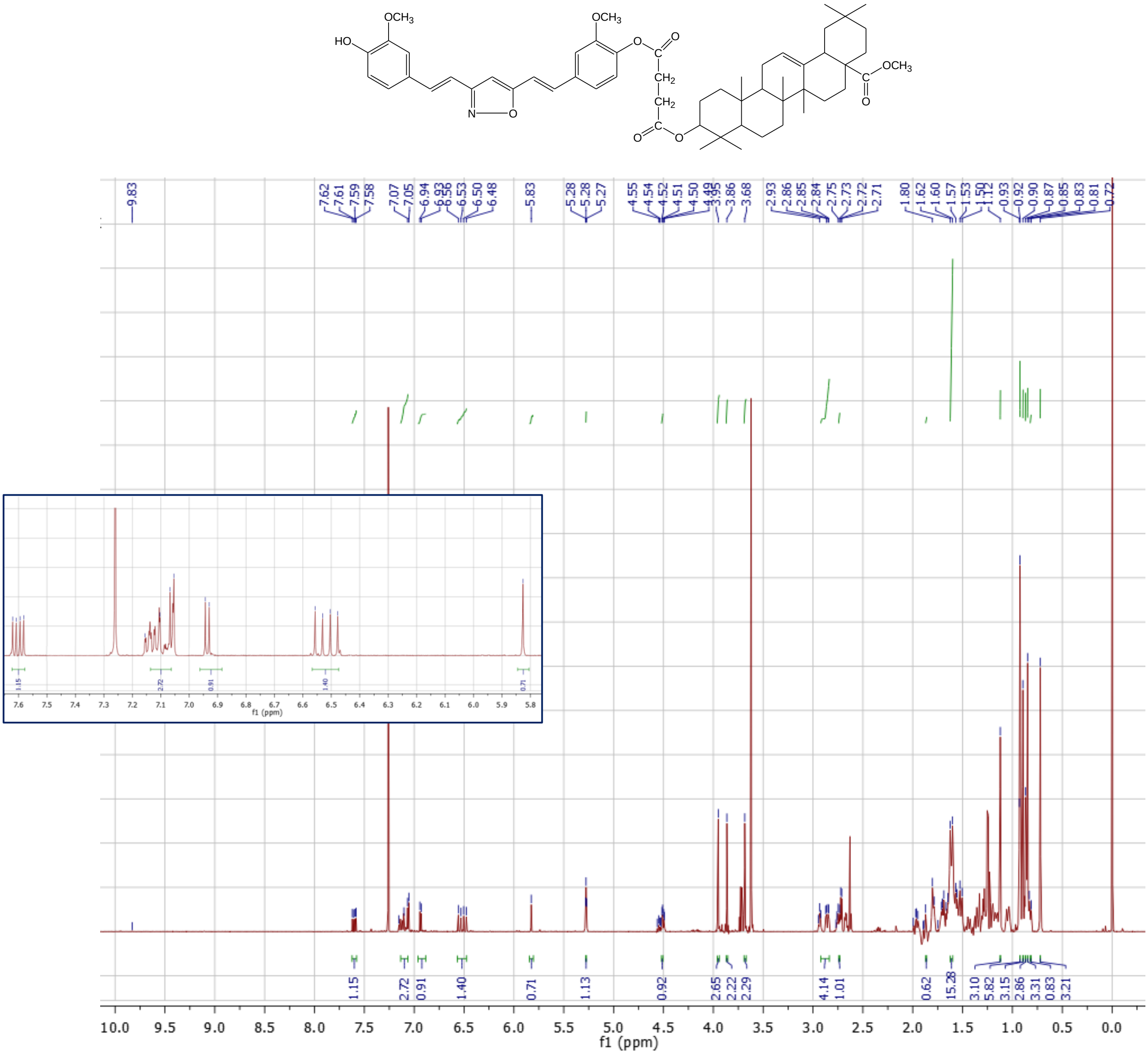

Figure S42 ^{1}H-NMR spectrum of Curcumin isoxazole mono-oleanoyl hydrogen succinate methyl ester

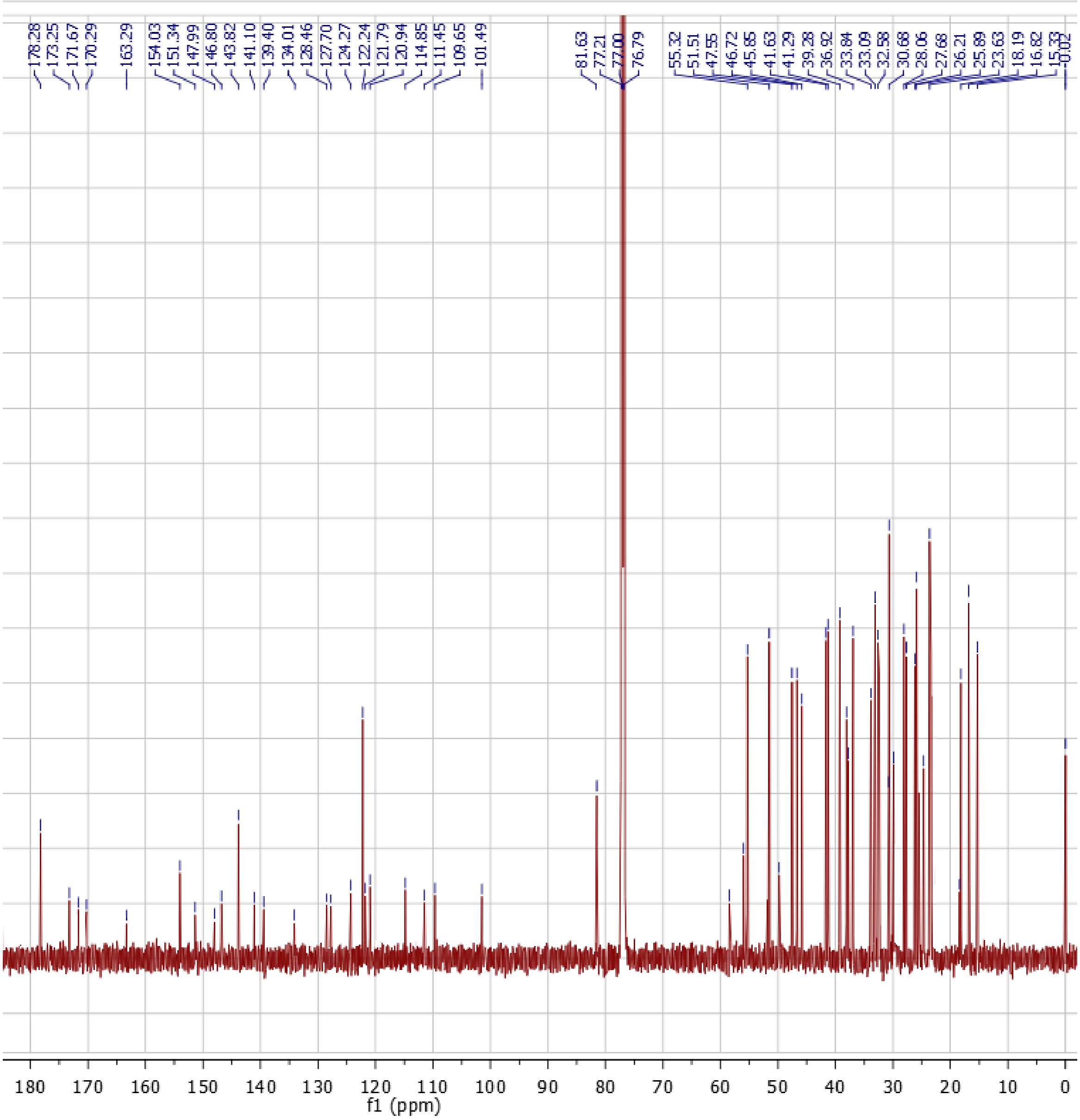

Figure S43 ^{13}C-NMR spectrum of Curcumin isoxazole mono-oleanoyl hydrogen succinate methyl ester
